# Using linked national registry data and the ECDC HIV modelling tool to estimate HIV incidence and the proportion diagnosed in Norway up to 2023

**DOI:** 10.1186/s12879-025-11541-x

**Published:** 2025-09-23

**Authors:** Robert Whittaker

**Affiliations:** Department of Infection Control and Vaccines, Norwegian Institute of Public Health, Lovisenberggata 8, Oslo, 0456 Norway

**Keywords:** HIV, Disease elimination, Public health surveillance, Epidemiological models, Norway, Electronic health records

## Abstract

**Background:**

Estimates of HIV incidence and the size of the undiagnosed population are important to monitor epidemic trends, assess progress towards regional and global goals, and inform ongoing prevention and control measures. Previous estimates from Norway date back to 2018 and did not account for pre-migration infection among migrants. This is essential when modelling the HIV epidemic in countries like Norway, where over 80% of new HIV diagnoses are among migrants who reportedly acquired HIV before migration.

**Method:**

I estimated the incidence of new HIV infections, number undiagnosed and proportion diagnosed overall and in key groups at risk of HIV acquisition up until the end of 2023 in Norway. I used linked national registry data and the ECDC HIV modelling tool, a back-calculation compartmental model that uses routine surveillance data on HIV diagnoses. The modelling tool estimates pre-migration infections by comparing the known date of migration to the estimated date of infection. I generated estimates overall and for four subgroups: Norwegian-born men who have sex with men (MSM), Norwegian-born heterosexual transmission, migrant MSM and migrant heterosexual transmission.

**Results:**

The model input data included 7,185 new HIV diagnoses up to the end of 2023, of which 4,266 (59%) were migrants. The model estimated that 86% (95% confidence interval (CI): 83% – 88%) of new infections among migrants were pre-migration infections. Excluding these, there were 13 (95% CI: 11 – 50) new HIV infections in Norway in 2023. There were 234 (95% CI: 207 – 336) undiagnosed PLHIV and the proportion diagnosed was 96.5% (95% CI: 95.1% – 96.9%). The diagnosed fraction was under 95% among Norwegian-born heterosexual transmission (92.6%; 95% CI: 87.1% – 94.5%) and migrant MSM (92.2%; 95% CI: 90.8% – 93.8%).

**Conclusions:**

Overall, Norway has achieved the global target of diagnosing 95% of people living with HIV. The incidence of new HIV infections is low. Existing measures should be maintained and strengthened to further reduce the undiagnosed population and prevent transmission. The lower proportion diagnosed among Norwegian-born persons infected through heterosexual transmission and migrant MSM indicates where larger relative improvements may be made.

**Supplementary Information:**

The online version contains supplementary material available at 10.1186/s12879-025-11541-x.

## Introduction

Human immunodeficiency virus (HIV) causes an infection that, if untreated, can lead to acquired immunodeficiency syndrome (AIDS). In 2023, an estimated 39 million people were living with HIV infection (PLHIV) worldwide, with 1.3 million new infections and 630,000 HIV-related deaths [1].

Under the sustainable development goals, member states of the World Health Organisation (WHO) have committed to ending AIDS as a public health threat by 2030 [2]. Two targets for achieving this in Europe include an absolute incidence of new HIV infections < 2 per 100,000 uninfected population (reflecting a 90% relative decrease, compared to 2010), and that ≥ 95% of PLHIV know their HIV status (diagnosed fraction) [2]. Monitoring progress towards these targets is important, both in the general population and key populations at increased risk of HIV infection, such as men who have sex with men (MSM) and migrants (persons born in another country) [3].

An empirical modelling approach is recommended to estimate incidence and undiagnosed PLHIV [3]. One of the recommended and most commonly used tools in Europe is the HIV modelling tool from the European Centre for Disease Prevention and Control (ECDC) [3, 4]. It uses routine surveillance data on notified cases of HIV infection and AIDS in a deterministic, back-calculation compartmental model to estimate new HIV infections over time and the number undiagnosed.

One recent development to the tool includes accounting for pre-migration infections, by comparing the known date of migration to the model estimated date of infection [4]. This allows the incidence of preventable new infections among persons born or resident in the country (‘endogenous infections’ [5]) to be more accurately estimated. This is important in countries like Norway, where most new HIV diagnoses are now among migrants who reportedly acquired HIV before migration [6]. In 2023, 17 European Union (EU) and European Economic Area (EEA) countries reported over 50% of new HIV diagnoses among migrants [7].

Furthermore, in the tool data on concurrent HIV/AIDS diagnoses (defined as an AIDS-defining diagnosis within 3 months of HIV diagnosis) are important for back-calculating the incidence curve from the date of diagnosis. Also, deaths and out-migrations among persons diagnosed with HIV are needed to estimate the number of PLHIV. Previous modelling in Norway (using the ECDC tool) only utilised routine HIV surveillance data [8], where clinical AIDS diagnoses, deaths and out-migrations are underreported [6, 8]. Similar challenges are present in surveillance data in other EU/EEA countries. For example, in 2023 only six countries reported that they could fully account for deaths and out-migration [3].

In this study, I used an enhanced dataset of linked national registry data and the ECDC HIV modelling tool to generate updated estimates of HIV incidence and the diagnosed fraction in Norway overall and in key groups at risk of HIV acquisition up until the end of 2023. I also compared results from modelling using this enhanced dataset to modelling when using data from routine HIV surveillance only.

## Methods

### Study setting

#### Public health response

As of 2023, the population in Norway was 5.5 million persons, 16% of whom were born outside Norway. Testing and health services for HIV are free of charge, regardless of legal status. A wide range of groups are recommended testing [9]. Several actors offer information, support and/or low threshold testing to key groups at risk of HIV acquisition [10], for example anonymous peer-to-peer rapid diagnostic testing for MSM since 2012 [11]. Refugees and asylum seekers from high-prevalence countries are recommended to be offered testing by healthcare services as part of a routine health check three months after arrival [12]. All pregnant women are offered testing in the first trimester. Since 2016, national clinical guidelines have recommended antiretroviral treatment of all persons diagnosed with HIV, regardless of CD4 count. Pre-exposure prophylaxis (PrEP) has been available and publicly funded since January 2017. The number of PrEP users has increased steadily, reaching 3,500 in 2024 [6]. This is among the highest rates per capita in the EU/EEA, although the PrEP need in Norway is uknown [7]. Needle and syringe programmes for people who inject drugs (PWID) have been available since 1987 and opioid agonist therapy since 1998. Coverage of these two harm reduction interventions exceeds global targets for the elimination of HIV and viral hepatitis as public health threats [2, 13].

#### Trend in HIV diagnoses

The HIV epidemic in Norway was initially characterised by diagnoses of HIV infection among MSM and PWID in the mid-1980s. Diagnoses peaked in the mid-2000s, driven by increased migration from high-endemic areas, such as sub-Saharan Africa, and an increase in diagnoses among MSM. From 2008 to 2021, there was a declining trend among both Norwegian-born MSM and migrants infected through heterosexual transmission. From 2020–2023, there were < 60 new HIV diagnoses annually among people reportedly resident in Norway at the time of HIV infection, with < 30 reportedly acquiring HIV in Norway. An increase in HIV diagnoses in 2022 and 2023 was driven by the arrival of 72,000 refugees from Ukraine [6].

Among persons newly diagnosed with HIV, the proportion born outside Norway has increased gradually from < 40% before the year 2000, to around 60% from 2000–2019 and 84% from 2019–2023. Up to 2023, over 80% of all new HIV diagnoses among migrants have been reported by the notifying clinician to have acquired HIV before migration to Norway. This proportion was 80% from 2011–2021, and 92% from 2022–2023. Further details are available in national surveillance reports [6].

### Data sources to identify new HIV diagnoses

#### Norwegian surveillance system for communicable diseases

In Norway, cases of HIV infection have been mandatorily notifiable by clinicians and laboratories to the Norwegian Surveillance System for Communicable Diseases (MSIS) since 1986. The case definition is in line with the EU definition [14]. Data were reported anonymously (without national identity number) until 22 March 2019. Data on CD4 count at diagnosis have also been registered since March 2019, with completeness increasing from 45% in 2019 to 76% in 2020 and 96–99% in 2021–2023.

I included all notified cases of HIV-1 infection from 1987–2023 with data on diagnosis date, age, sex, mode of transmission, country of birth, known previous positive test before diagnosis, concurrent AIDS at HIV diagnosis, CD4 count, and death and out-migration.

#### Norwegian patient registry

Notified cases of HIV infection with a national identity number were only available in MSIS from 2019. To get a linkable cohort of persons with newly diagnosed HIV prior to 2019, I used the Norwegian Patient Registry (NPR). In Norway, linkage to specialist care after HIV diagnosis is high, with treatment uptake reportedly 95% – 98% [15, 16]. NPR contains data on all inpatient and outpatient hospital stays, with national identifiers collected for all patients since 2008.

I identified patients with somatic hospital stays from 2011–2018 with an International Classification of Diseases, 10th revision (ICD-10) code for HIV (B20 – B24 or Z21) registered at least twice. I included data on consultation date, age, sex and ICD-10 code. The supplement, part 1 further details the choice of this sample and compares new HIV diagnoses in MSIS and NPR over time.

### Model input dataset

The model dataset included HIV diagnoses from MSIS from 1987–2010, NPR from 2011–2018 and MSIS from 2019–2023. The start year (1987) was chosen to avoid the early peak in HIV diagnoses before this year, related to the introduction of HIV testing [8].

For people diagnosed with HIV who had a linkable national identity number registered (NPR: 2011–2018, 100% linkable; MSIS: 2019–2023, 82% linkable), I supplemented these with AIDS notifications from MSIS (reported nominatively and separately from HIV notifications to MSIS since 1983 [6, 14]) and ICD-10 codes for specific AIDS-defining illnesses in NPR. These ICD-10 codes and a comparison to AIDS notifications in MSIS are presented in the supplement, part 2. I also included data on the date of migration to Norway, residence status (for example, resident, died, out-migrated) and country of birth from the National Population Register [17].

Table 1 presents key data points in the model dataset and compares these to data from routine HIV surveillance data alone. Data on date of HIV diagnosis, age and sex was known for all people diagnosed with HIV. Country of birth was unknown for three. The model dataset had a higher number of people diagnosed with AIDS (808 vs. 593) and deaths/out-migrations (904 vs. 614) than routine HIV surveillance data alone. Among persons born outside Norway and diagnosed with HIV from 2011–2023, 84% had known date of migration.

**Table 1:** Model input data and comparison to data from routine HIV surveillance data alone

Data point	Model input data			Routine surveillance data		
	**Data source**	**Year of data point**	**N**	**Data source**	**Year of data point**	**N**
HIV diagnoses	MSIS	1987–2010	4,191	MSIS	1987–2010	4,191
	NPR	2011–2018	2,006		2011–2018	1,835
	MSIS	2019–2023	988		2019–2023	988
Known CD4 count at HIV diagnosis^a^	MSIS	2019–2023	835	MSIS	2019–2023	835
AIDS diagnoses	MSIS MSIS and NPR	1987–2010	359	MSIS	1987–2010	359
		2011–2018	293		2011–2018	153
		2019–2023	155		2019–2023	81
Deaths	MSIS and Freg	1987–2010	477	MSIS	1987–2010	477
		2011–2023	149		2011–2023	18
Out-migrations	MSIS and Freg	1987–2010 2011–2023	103^b^ 173	MSIS	1987–2010 2011–2023	90 29
Born outside Norway	MSIS and Freg	1987–2010	2,228	MSIS	1987–2010	2,228
		2011–2018	1,234		2011–2018	1,153
		2019–2023	804		2019–2023	804
Born outside Norway and known to have been previously diagnosed with HIV before arrival to Norway	MSIS^c^	1987–2010	265	MSIS	1987–2023	1,142
		2019–2023	524			
Born outside Norway and with known date of migration	Freg	2011–2023	1,716	^d^	^d^	^d^

*Freg* National Population Register, *MSIS* Norwegian Surveillance System for Communicable Diseases, *NPR* Norwegian Patient Registry

^a^Reporting of data on CD4 count in Norway started on 22 March 2019

^b^The discrepancy between the model input data and the routine data for 1987–2010 (i.e. the period before the linked data were available) is because 13 people diagnosed with HIV identified in NPR were registered as having out-migrated in the years 1987–2010, but were diagnosed with HIV in Norway from 2011–2018, and thus may be been diagnosed on a visit to Norway

^c^100% unknown for 2011–2018, as these data are not available in NPR

^d^Date of migration unavailable in the routine dataset

### Application of ECDC HIV modelling tool

I used the ECDC HIV modelling tool version 3.1.5 (3 April 2025), modelling from 1980–2023. I ran models for all PLHIV, persons born in Norway and migrants. I also modelled the four subgroups that constitute the majority of new HIV diagnoses in Norway [6] (Norwegian-born MSM, Norwegian-born heterosexual transmission, migrant MSM, migrant heterosexual transmission). I also ran models utilising routine HIV surveillance data from MSIS only, to explore how results varied, compared to the enhanced model dataset. Pre- and post-migration infections were estimated from 2011–2023. Further details on the input parameters, diagnosis matrix (which determines the shape of the diagnosis probability) and goodness of fit are presented in the supplement, parts 3, 4 and 5. Further details on the underlying model method are available in the model manual [4].

I reported on estimates generated for the incidence of new HIV infections, the number of PLHIV, the number of undiagnosed PLHIV and the diagnosed fraction. For incidence I described the estimated total number of new infections and the number of endogenous infections (i.e. excluding pre-migration infections). I used 100 non-parametric bootstrap iterations to determine 95% confidence intervals (95% CI), defined by the 2.5% and 97.5% percentiles.

## Results

### Overall

**Fig. 1:**
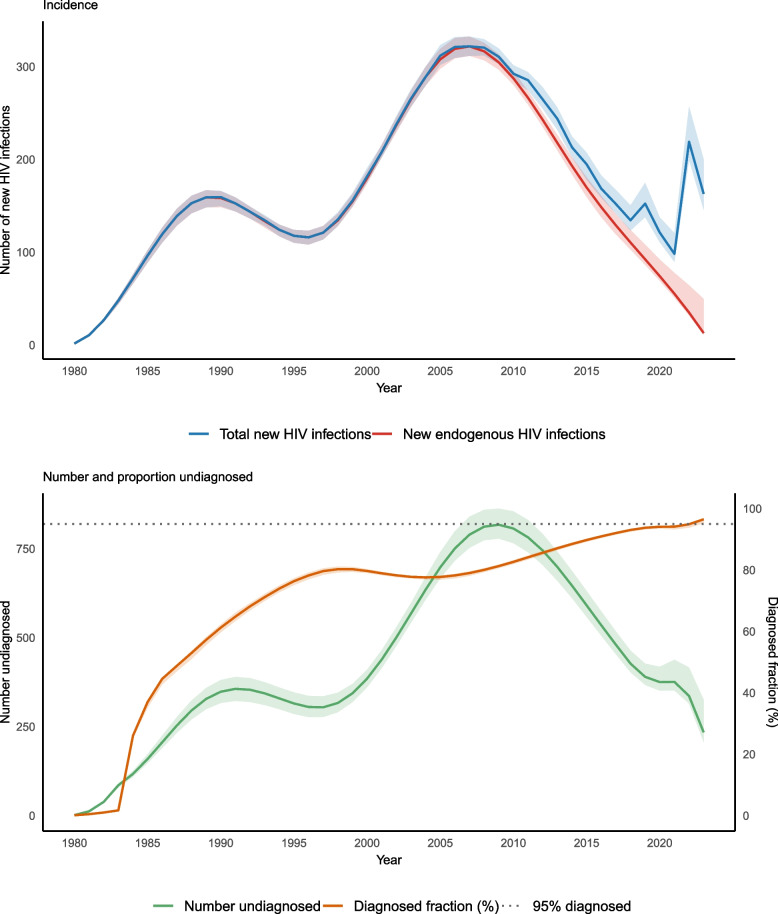
Estimated number of new HIV infections and undiagnosed infections, Norway, 1980–2023 Endogenous infections: infections among persons born or resident in Norway. As data on the date of migration was not available for persons diagnosed with HIV before 2011, it was not possible to distinguish endogenous infections from estimates of all new HIV infections before this year. The dotted line for 95% diagnosed indicates the UNAIDS goal of 95% of people living with HIV being diagnosed by 2025. The shaded area around the estimates line reflects the 95% confidence interval

Incidence peaked at 322 (95% CI: 312–332) new HIV infections in 2007 and thereafter decreased steadily, reaching 13 (95% CI: 11–50) endogenous infections in 2023 (Fig. 1, Table 2). This reflects a 95% (83% – 96%) decrease, compared to the estimated incidence in 2010 (287 (95% CI: 281–295)). New endogenous infections accounted for 7.9% (95% CI: 7.7% – 25%) of the estimated 163 (95% CI: 146–200) total new infections (i.e. including pre-migration infections) in 2023 (Fig. 1).

**Table 2:** Number of diagnosed cases of HIV infection in model input data and key model outputs for 2023

Population^a^	Number of diagnosed cases of HIV in model input data (%)	Model outputs			
		**Number of new endogenous HIV infections**^**b**^** in 2023 (95%CI)**	**Number of PLHIV in 2023 (95%CI)**	**Number of undiagnosed PLHIV in 2023 (95%CI)**	**Proportion diagnosed in 2023 (95%CI)**
All PLHIV	7,185 (100%)	13 (11–50)	6,726 (6,683–6,856)	234 (207–336)	96.5 (95.1–96.9)
By country of birth					
Norwegian-born	2,919 (41%)	5 (3–67)	2,645 (2,549–2,844)	109 (75–289)	95.9 (89.9–97.1)
Migrants	4,266 (59%)	9 (8–36)	4 098 (4,030–4,189)	128 (103–183)	96.9 (95.6–97.4)
By country of birth and mode of transmission					
Norwegian-born MSM	1,511 (21%)	7 (1–30)	1,357 (1 294–1 434)	47 (17–110)	96.6 (92.3–98.7)
Norwegian-born heterosexual transmission	968 (12%)	3 (2–24)	937 (904–1,040)	69 (50–132)	92.6 (87.1–94.5)
Migrant MSM	862 (13%)	4 (4–6)	841 (796–881)	65 (49–81)	92.2 (90.2–94.2)
Migrants heterosexual transmission	2,924 (41%)	3 (2–14)	2,804 (2,751–2,886)	68 (49–101)	97.6 (96.5–98.2)

* CI* Confidence interval, *MSM* Men who have sex with men, *PLHIV* People who live with HIV

^a^People who inject drugs are an important group at risk of HIV acquisition, however were not modelled separately, as the number of new diagnoses in Norway is low (< 10 per year from 2015–2024) [6] and previous modelling [8] and prevalence studies [9] have found low incidence and few undiagnosed infections

^b^Endogenous infections: infections among persons born or resident in Norway

The number of undiagnosed PLHIV decreased steadily from a peak of 817 (95% CI: 778–863) in 2009 to 234 (95% CI: 207–336) in 2023 (Fig. 1). The point estimate of the diagnosed fraction first exceeded 95% in 2023 (96.5%, 95% CI: 95.1% – 96.9%) (Fig. 1, Table 2).

When the model was run on routine HIV surveillance data from MSIS only, estimated incidence in 2023 was 196 (95% CI: 152–251) new infections, with 347 (95% CI: 258–524) undiagnosed PLHIV and a diagnosed fraction of 95.0% (95% CI: 92.6% – 96.2%). Further results from models run on these input data are presented in the supplement, part 6.

### Persons born in Norway

**Fig. 2:**
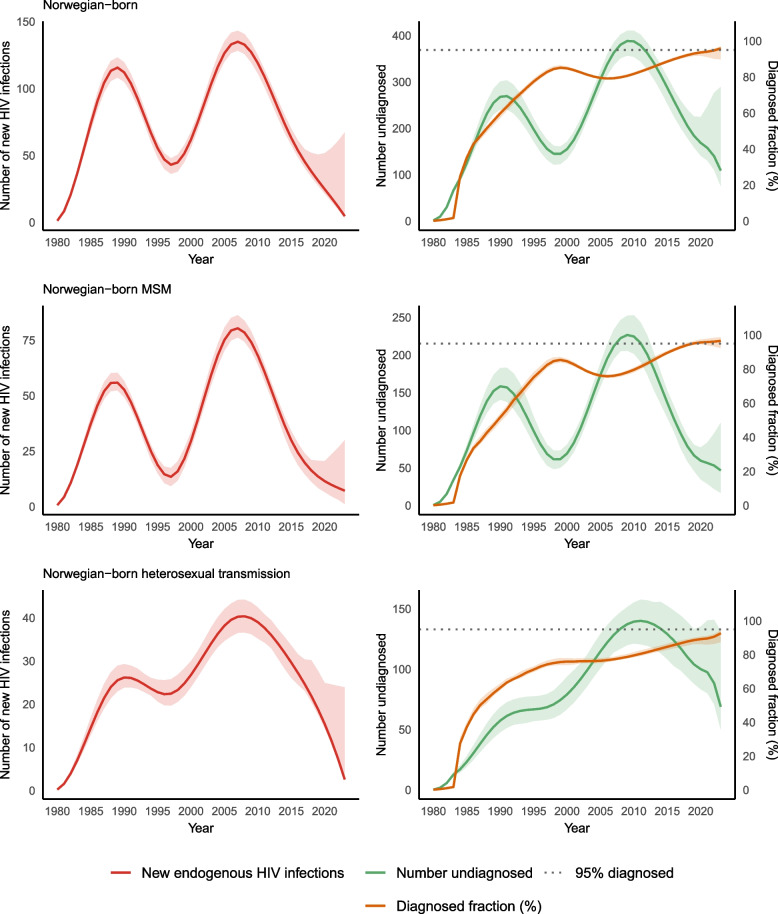
Estimated number of new HIV infections and undiagnosed infections among Norwegian-born persons, Norway, 1980–2023 MSM: Men who have sex with men. Endogenous infections: infections among persons born or resident in Norway. Thus, for persons born in Norway, all new infections are endogenous infections. The dotted line for 95% diagnosed indicates the UNAIDS goal of 95% of people living with HIV being diagnosed by 2025. The shaded area around the estimates line reflects the 95% confidence interval

Incidence peaked at 135 (95% CI: 128–143) new infections in 2007. It thereafter decreased steadily to 5 (95% CI: 3–67) in 2023 (Fig. 2, Table 2), a 96% (46% – 97%) relative decrease, compared to the estimated incidence in 2010 (119 new infections, 95% CI: 111–125). The number of undiagnosed PLHIV decreased from a peak of 388 (95% CI: 357–410) in 2009 to 109 (95% CI: 75–289) in 2023, a diagnosed fraction of 95.9% (95% CI: 89.9–97.1) (Fig. 2). The diagnosed fraction was lower among those who acquired HIV through heterosexual transmission (92.6%; 95% CI: 87.1% – 94.5%) than MSM (96.6%; 95% CI: 92.3% – 98.7%) (Table 2, Fig. 2). The point estimate for the diagnosed fraction among MSM first exceeded 95% in 2020.

### Migrants to Norway

The model estimated that 86% (95% CI: 83% – 88%) of new HIV infections among migrants from 2011 were acquired prior to arrival, with the point estimate hovering around 20% from 2011–2021 and 10% in 2022 and 2023. The proportion was lower among MSM (78%, 95% CI: 71% – 83%) than other modes of transmission (see the supplement, part 7).

**Fig. 3:**
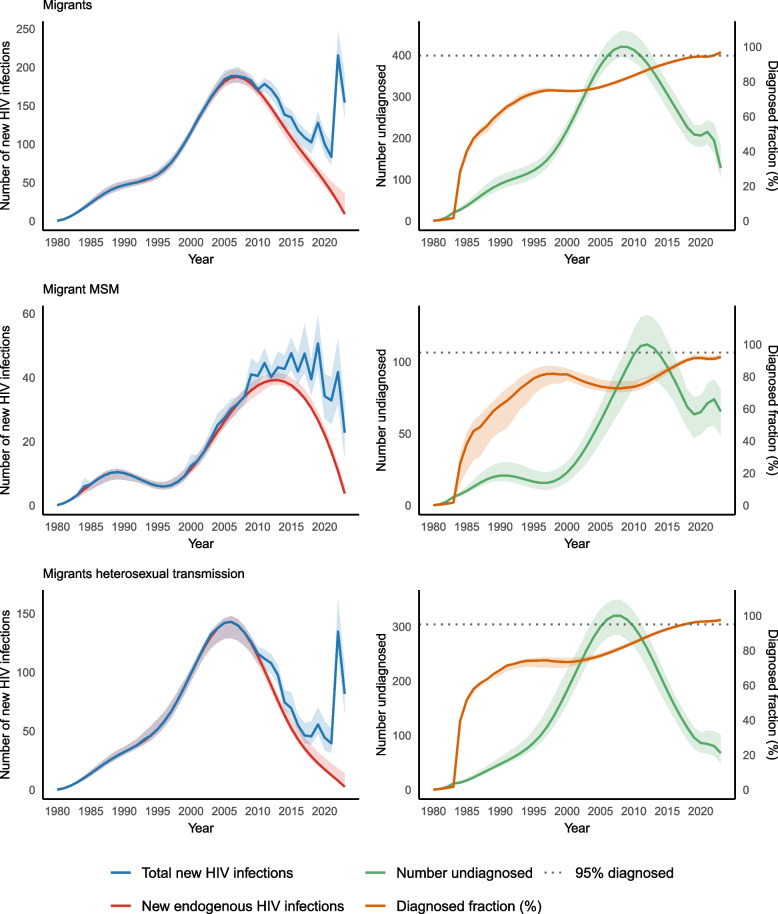
Estimated number of new HIV infections and undiagnosed infections among migrants, Norway, 1980–2023 MSM: Men who have sex with men. Endogenous infections: infections among persons born or resident in Norway. As data on the date of migration was not available for persons diagnosed with HIV before 2011, it was not possible to distinguish endogenous infections from estimates of all new HIV infections before this year. The dotted line for 95%nullnull diagnosed indicates the UNAIDS goal of 95% of people living with HIV being diagnosed by 2025. The shaded area around the estimates line reflects the 95% confidence interval

Incidence peaked at 188 (95% CI: 180–197) new infections in 2007 and thereafter decreased steadily to 9 (95% CI: 8–36) endogenous infections in 2023, 6.0% (95% CI: 5.6% – 19.0%) of the estimated 154 (95% CI: 134–190) total new infections that year (Fig. 3). The number of undiagnosed PLHIV decreased from a peak of 420 (95% CI: 394–459) in 2009 to 128 (95% CI: 103–183) in 2023, a diagnosed fraction of 96.9% (95% CI: 95.6–97.4) (Fig. 3). The diagnosed fraction was lower among MSM than (92.2%; 95% CI: 90.8% – 93.8%) than those who acquired HIV through heterosexual transmission (97.6%; 95% CI: 96.5% – 98.2%), with a similar number of undiagnosed PLHIV (Table 2, Fig. 3). The point estimate for the diagnosed fraction among migrants who acquired HIV through heterosexual transmission first exceeded 95% in 2018.

## Discussion

In this study, I used an enhanced dataset of linked national registry data and the ECDC HIV modelling tool to generate updated estimates of HIV incidence and the diagnosed fraction for Norway. The linked dataset allowed pre-migration infections, underreporting of concurrent HIV/AIDS diagnoses, and out-migrations and deaths among diagnosed PLHIV to be considered from 2011 onwards. The added value of this linkage is clear when comparing results to modelling using routine HIV surveillance data form MSIS only, which resulted in a notable overestimation in incidence and uncertainty over whether the goal to diagnose > 95% of PLHIV had been achieved.

Results suggest that the incidence of new HIV infections and number of undiagnosed PLHIV in Norway have decreased consistently for more than a decade, and that the absolute incidence level is now below the European target of < 2 per 100,000 population [2]. The model estimated annual proportion of pre-migration infections in Norway averaged 86% (95% CI: 83% – 88%), slightly higher than the average in Europe of around 70% [18, 19] and suggesting a low level of HIV transmission among migrants after arrival.

Results also suggest that Norway has now achieved the goal of diagnosing > 95% of PLHIV. According to ECDC summaries, this makes Norway the fifth EU/EEA country to document achievement of all three stages of the UNAIDS 95–95-95 goals (at least 95% diagnosed, 95% on treatment and 95% successfully treated), alongside Denmark, Finland, Iceland and Sweden [5, 7]. Some fast-track cities have also documented this achievement [20]. Norway is also the third EU/EEA country to document achieving the first 95% target among migrants, although only six other countries can report such estimates [7], highlighting an important regional data gap.

The relative decrease in incidence compared to 2010 is more difficult to assess. Data linkage was only available from 2011 onwards so pre-migration infection is not considered before 2011 and estimates for any models including migrants for these years likely overestimated. In Sweden, a country with a similar HIV epidemic to Norway, around half of all new HIV infections form 2003–2011 were estimated to have been pre-migration [5]. This may give some idea as to how much pre-2011 incidence is overestimated in my model. Point estimates among Norwegian-born PLHIV, not affected by migration, suggest that a reduction of > 90% has been achieved. Yet uncertainty remains here as well as 95% CI were wide at the end of the modelled period, a known phenomenon with back-calculation models like the ECDC tool [20].

Overestimates of incidence and incomplete data on out-migrations and deaths from the earlier part of the epidemic will also influence estimates at the end of the modelled period. An example is the PLHIV estimate of 6,726 in 2023. In Norway, most people diagnosed with HIV are retained in specialist care [15, 16]. However, as of the end of 2023, only around 4,900 individuals who had attended a consultation in specialist care for HIV were registered as still resident in Norway [6]. Considering also the estimated number of undiagnosed PLHIV in this study, it is therefore more likely that there were around 5,100 PLHIV in Norway at the end of 2023. However, given that overestimates of incidence will also result in a higher estimated number undiagnosed, I can still be confident in concluding that Norway has achieved the goal of 95% of PLHIV being diagnosed, even with a lower denominator for PLHIV.

The results underline the effect of the wider public health response to HIV in Norway, as described in the study setting. Yet, there remain approximately 200–300 undiagnosed PLHIV and new infections are still estimated in all modelled subgroups. Also, while the number of new HIV diagnoses among people resident in Norway at the time of infection has remained stably low over the past few years, the proportion diagnosed late (CD4 count below 350 or AIDS-defining event) [21] has remained around 30% – 40% and was over 50% among notified cases who acquired HIV through heterosexual sex [6]. Thus, the European goal of < 20% diagnosed late by 2025 has not be achieved in Norway [2], reflecting the general trend in the EU/EEA [22]. Measures to ensure HIV prevention, prompt diagnosis and adequate clinical follow-up must therefore be maintained and strengthened. While the estimated number of undiagnosed PLHIV was similar across the modelled subgroups, there was a lower diagnosed fraction among Norwegian-born persons infected through heterosexual contact and migrant MSM. Also, a higher proportion of migrant MSM were estimated to have acquired HIV after migration. These results indicate where larger relative improvements may be made.

In Norway, relevant measures to further reduce the undiagnosed population and prevent transmission mirror those described elsewhere in the EU/EEA [20, 23–26], including ensuring a low barrier to testing for key groups at risk of HIV acquisition, sufficient contact tracing following new diagnoses and that persons presenting to health care with indicator conditions for HIV testing are offered testing. High treatment uptake must be maintained and PrEP offered to those with an indication [27]. High coverage of other primary preventative measures, including condoms, and needle and syringe programmes, is also essential. For groups at higher risk of HIV acquisition, the health care system must be easy to reach and navigate, and integrated and differentiated models of care should be tailored to groups’ specific needs. Such services must be delivered free of stigma and discrimination, which remains a challenge both in Norway and across the EU [23, 28, 29]. Awareness and knowledge of HIV must be maintained and increased among health care professionals and groups at higher risk of acquiring HIV. Such services for migrants will remain of particular importance for the foreseeable future, as global HIV incidence reduction has been slow (20% from 2011–2021) and the number of PLHIV is predicted to continue to increase through the 2030 s [30].

My study has several strengths. National data were available from the start of the epidemic and data completeness for important input variables, such as age, sex, data of migration and CD4 count, was high for years in which these data were available. The data linkage provides a framework for future modelling of the HIV epidemic in Norway and may soon be incorporated into routine surveillance data flows [31]. Results also highlight the value of the ECDC tool, which makes estimating key indicators of the HIV epidemic and progress towards strategic goals accessible, especially in countries like Norway, where the development of more advanced bespoke models, for example using negative test results and biomarkers other than CD4 count [5, 32], is currently challenging, given data availability. The national clinical registry for HIV in Norway is developing and as of 2023 included about 40% of diagnosed PLHIV [16]. Negative test results are collected in the national laboratory database, although legal restrictions currently prevent these from being linked to other registries [33].

There are also some important limitations with my study. Some limitations with data linkage only being available from 2011 onwards are mentioned above. Also, the ECDC model may not correctly identify pre-migration or post-migration infection in an estimated 10% – 20% of HIV diagnoses [18]. However, the model estimated annual proportion of pre-migration infections closely matched the proportion reported by notifying clinicians to have acquired HIV before migration to Norway in routine surveillance data. Furthermore, I do not know how well the selected ICD-10 codes in NPR reflect true AIDS diagnoses, however the codes selected were specific for AIDS-defining illnesses and did not cover all indicator conditions, so may still have underestimated HIV/AIDS diagnoses. Finally, it is unknown how well internal model assumptions on HIV progression that estimate infection from diagnosis dates apply to PLHIV in Norway.

## Conclusions

Norway has achieved the WHO goals for absolute HIV incidence and the diagnosed fraction. Existing measures should be maintained and strengthened to further reduce the undiagnosed population and prevent transmission. The methodology provides a framework for future modelling of the HIV epidemic in Norway.

## Supplementary Information

Supplementary Material 1

## Data Availability

The dataset analysed in the study contains individual-level data from national registries in Norway. Anyone is freely able to apply for access to data from the same registries for research purposes, as per normal procedure for conducting health research on registry data in Norway. Detailed results for all models are available on request.

## Abbreviations

HIV: Human immunodeficiency virus
PLHIV: People who live with HIV infection
AIDS: Acquired immunodeficiency syndrome
WHO: World Health Organisation
MSM: Men who have sex with men
ECDC: European Centre for Disease Prevention and Control
EU: European Union
EEA: European Economic Area
PrEP: Pre-exposure prophylaxis
PWID: People who inject drugs
MSIS: Norwegian Surveillance System for Communicable Diseases
NPR: Norwegian Patient Registry
ICD-10: International Classification of Diseases, 10th revision
CI: Confidence Interval

## References

[1] Joint United Nations Programme on HIV/AIDS (UNAIDS). 2024 global AIDS report — the Urgency of Now: AIDS at a Crossroads. Geneva: UNAIDS; 2024. Available from: https://www.unaids.org/sites/default/files/media_asset/2024-unaids-global-aids-update_en.pdf.

[2] World Health Organization (WHO) Regional Office for Europe. Regional action plans for ending AIDS and the epidemics of viral hepatitis and sexually transmitted infections 2022–2030. Copenhagen: WHO; 2023. Available from: https://apps.who.int/iris/bitstream/handle/10665/369243/9789289058957-eng.pdf?sequence=7&isAllowed=y.

[3] European Centre for Disease Prevention and Control (ECDC). Continuum of HIV care: Monitoring implementation of the Dublin Declaration on partnership to fight HIV/AIDS in Europe and Central Asia: 2023 progress report. Stockholm: ECDC; 2024. Available from: https://www.ecdc.europa.eu/sites/default/files/documents/hiv-dublin-continuum-care-progress-report-2023.pdf.

[4] ECDC. HIV platform tool. Stockholm: ECDC; 2021. Available from: https://www.ecdc.europa.eu/en/publications-data/hiv-platform-tool.

[5] Lundgren E, Locke M, Romero-Severson E, Dimitrijevic M, Axelsson M, Andersson E, et al. Sweden surpasses the UNAIDS 95–95–95 target: estimating HIV-1 incidence, 2003 to 2022. Euro Surveill. 2024;29(42). 10.2807/1560-7917.ES.2024.29.42.2400058. 10.2807/1560-7917.ES.2024.29.42.2400058 PMC11487918 39421951

[6] Norwegian Institute of Public Health. Blod- og seksuelt overførbare infeksjoner i Norge: Årsrapport 2024. [Blood and sexually transmitted infections in Norway: annual report 2024]. Oslo: Norwegian Institute of Public Health; 2025. Available from: https://www.fhi.no/publ/2025/arsrapport-2024---blod--og-seksuelt-overforbare-infeksjoner/.

[7] ECDC. Progress towards reaching the sustainable development goals related to HIV in the European Union and European Economic Area. Monitoring implementation of the Dublin Declaration on partnership to fight HIV/AIDS in Europe and Central Asia – 2024 progress report. Stockholm: ECDC; 2024. Available from: https://www.ecdc.europa.eu/sites/default/files/documents/hiv-evidence-brief-progress-towards-sustainable-development-goals-2023.pdf.

[8] Whittaker R, Case KK, Nilsen O, Blystad H, Cowan S, Kløvstad H, et al. Monitoring progress towards the first UNAIDS 90–90-90 target in key populations living with HIV in Norway. BMC Infect Dis. 2020;20(1):451. 32590964 10.1186/s12879-020-05178-1 PMC7318482

[9] Norwegian Institute of Public Health. Hivinfeksjon/Aids – håndbok for helsepersonell. [HIV/AIDS – handbook for health care professionals]. Oslo: Norwegian Institute of Public Health; 2025. Available from: https://www.fhi.no/sm/smittevernhandboka/sykdommer-a-a/hivinfeksjonaids/?term=. Accessed: 09 June 2025.

[10] Helse Norge. Rapid HIV test. Oslo: Helse Norge; 2024. Available from: https://www.helsenorge.no/en/sykdom/hiv-aids/rapid-HIV-test/. Accessed: 09 June 2025.

[11] Helseutvalget. Sjekkpunkt. Oslo: Helseutvalget; 2025. Available from: https://www.helseutvalget.no/en/sjekkpunkt. Accessed: 09 June 2025.

[12] The Norwegian Directorate of Health. Helsetjenester til asylsøkere, flyktninger og familiegjenforente. [Healthcare services for asylum seekers, refugees and their family members]. Oslo: The Norwegian Directorate of Health; 2024. Available from: https://www.helsedirektoratet.no/veiledere/helsetjenester-til-asylsokere-flyktninger-og-familiegjenforente. Accessed: 09 June 2025.

[13] Whittaker R, Midtbø JE, Klovstad H. Monitoring progress towards the elimination of hepatitis C as a public health threat in Norway: a modelling study among people who inject drugs and immigrants. J Infect Dis. 2024;230(3):e700–11. 10.1093/infdis/jiae147. 38537267 10.1093/infdis/jiae147 PMC11420790

[14] Norwegian Institute of Public Health. Meldingskriterier for sykdommer i msis. [Case definitions for notifiable diseases]. Oslo: Norwegian Institute of Public Health; 2025. Available from: https://www.fhi.no/publ/informasjonsark/meldingskriterier-for-sykdommer-i-msis/. Accessed: 09 June 2025.

[15] Whittaker R, Nilsen O, Myrberg AJ, Angeltvedt RM, Bergersen BM, Kløvstad H. Norway is on the verge of ending the HIV epidemic. Tidsskr Nor Laegeforen. 2020;140(18). 10.4045/tidsskr.20.0748. 10.4045/tidsskr.20.0748 33322887

[16] Norsk kvalitetsregister for HIV. Årsrapport for 2023. [Annual report for 2023]. Oslo: University Hospital and the Norwegian Institute of Public Health; 2024. Available from: https://www.kvalitetsregistre.no/4acc55/siteassets/dokumenter/arsrapporter/hivregisteret/arsrapportnorhiv_2023_oppdatert.pdf.

[17] The Norwegian Tax Administration. National population register. Available from: https://www.skatteetaten.no/en/person/national-registry/. Accessed: 09 June 2025.

[18] Pantazis N, Rosinska M, van Sighem A, Quinten C, Noori T, Burns F, et al. Discriminating between premigration and postmigration HIV acquisition using surveillance data. J Acquir Immune Defic Syndr. 2021;88(2):117–24. 34138772 10.1097/QAI.0000000000002745

[19] Mann S, Mougammadou Z, Wohlfahrt J, Elmahdi R. Post-migration HIV acquisition: a systematic review and meta-analysis. Epidemiol Infect. 2024;152:e49. 38425215 10.1017/S0950268824000372 PMC11022255

[20] HIV Transmission Elimination AMsterdam (H-TEAM) Initiative. A 95% decline in estimated newly acquired HIV infections, Amsterdam, 2010 to 2022. Euro Surveill. 2023;28(40). 10.2807/1560-7917.ES.2023.28.40.2300515. 10.2807/1560-7917.ES.2023.28.40.2300515 PMC10557385 37796442

[21] Croxford S, Stengaard AR, Brannstrom J, Combs L, Dedes N, Girardi E, et al. Late diagnosis of HIV: an updated consensus definition. HIV Med. 2022;23(11):1202–8. 36347523 10.1111/hiv.13425 PMC10100195

[22] Reyes-Urueña J, Marrone G, Noori T, Kuchukhidze G, Martsynovska V, Hetman L, et al. HIV diagnoses among people born in Ukraine reported by EU/EEA countries in 2022: impact on regional HIV trends and implications for healthcare planning. Euro Surveill. 2023;28(48):2300642. 38037726 10.2807/1560-7917.ES.2023.28.48.2300642 PMC10690861

[23] Bremer V, Pharris A. Five years to 2030: reaching underserved populations is key to ending the AIDS epidemic in Europe. Euro Surveill. 2024;29(48). 10.2807/1560-7917.ES.2024.29.48.2400778. 10.2807/1560-7917.ES.2024.29.48.2400778 PMC11605800 39611204

[24] Parczewski M, Gokengin D, Sullivan A, de Amo J, Cairns G, Bivol S, et al. Control of HIV across the WHO European region: progress and remaining challenges. Lancet Reg Health. 2025;52:101243. 10.1016/j.lanepe.2025.101243 PMC11889346 40060938

[25] Isosomppi S, Mutru M, Ollgren J, Brummer-Korvenkontio H, Liitsola K, Sutinen J, et al. Use of healthcare services preceding HIV diagnosis - missed opportunities for earlier diagnosis, Finland, 1996 to 2019. Euro Surveill. 2025;30(18). 10.2807/1560-7917.ES.2025.30.18.2400610. 10.2807/1560-7917.ES.2025.30.18.2400610 PMC12066979 40341103

[26] Martinez Martinez V, Ormel H, de Op Coul ELM. Barriers and enablers that influence the uptake of HIV testing among heterosexual migrants in the Netherlands. PLoS ONE. 2024;19(10):e0311114. 39383170 10.1371/journal.pone.0311114 PMC11463776

[27] Norwegian Medical Association. Faglige retningslinjer for oppfølging og behandling av hiv i Norge. [National guidelines for the follow-up and treatment of HIV in Norway]. Oslo: Norwegian Medical Association; 2024. Available from: https://hivfag.no/images/2024/hivretningslinjer2024.pdf.

[28] HIV Norge. Hiv og stigma i helsevesenet. [HIV and stigma in healthcare]. Oslo: HIV Norge; 2024. Available from: https://hivnorge.no/nyheter/hiv-og-stigma-i-helsevesenet/#:~:text=En%20europeisk%20unders%C3%B8kelse%20viser%20at,overf%C3%B8ring%20og%20forebygging%20av%20hiv. Accessed: 09 June 2025.

[29] Krankowska DC, Lourida P, Quirke SM, Woode Owusu M, Weis N, Group EWMWW. Barriers to HIV testing and possible interventions to improve access to HIV healthcare among migrants, with a focus on migrant women: results from a European survey. HIV Med. 2024;25(5):554–64. 38197547 10.1111/hiv.13606

[30] GBD 2021 HIV Collaborators. Global, regional, and national burden of HIV/AIDS, 1990–2021, and forecasts to 2050, for 204 countries and territories: the Global Burden of Disease Study 2021. Lancet HIV. 2024;11(12):e807-e22. 10.1016/S2352-3018(24)00212-1 PMC11612058 39608393

[31] Norwegian Institute of Public Health. NORSURV. Oslo: Norwegian Institute of Public Health; 2024. Available from: https://www.fhi.no/en/projects/norsurv/. Accessed: 09 June 2025.

[32] McDonald SA, Yeung A, Nandwani R, Clutterbuck D, Wallace LA, Cullen BL, et al. A statistical model for inference of recent and incident HIV infection using surveillance data on individuals newly diagnosed with HIV infection in Scotland. J Acquir Immune Defic Syndr. 2024;97(2):117–24. 39250645 10.1097/QAI.0000000000003479

[33] Norwegian Institute of Public Health. Elektronisk laboratoriemelding til MSIS og laboratoriedatabasen. [Electronic laboratory notification to MSIS and the laboratory database]. Oslo: Norwegian Institute of Public Health; 2023. Available from: https://www.fhi.no/hn/helseregistre-og-registre/msis/elektronisk-laboratoriemelding-til-msis-og-laboratoriedatabasen/. Accessed: 09 June 2025.

