# Supplementary material for: Using linked national registry data and the ECDC HIV modelling tool to estimate HIV incidence and the proportion diagnosed in Norway up to 2023
Source: BMC Infect Dis. 2025 Sep 23;25:1115. doi: 10.1186/s12879-025-11541-x (PMC12455803; doi:10.1186/s12879-025-11541-x)
Supplement: Supplementary file 1 — Supplementary Material 1 [file 12879_2025_11541_MOESM1_ESM.docx]

**Using linked national registry data and the ECDC HIV modelling tool to estimate HIV incidence and the proportion diagnosed in Norway up to 2023.**

**Supplement**

**Contents**

[**1.** **Comparison of new HIV diagnoses over time in MSIS and NPR** 2](#_Toc205294704)

[**2.** **Defining AIDS based on NPR data and comparison to AIDS cases notified in MSIS** 5](#_Toc205294705)

[**3.** **Additional details on model inputs and parameters** 8](#_Toc205294706)

[**4.** **Diagnosis matrix and goodness-of-fit** 9](#_Toc205294707)

[**5.** **Alternative diagnosis matrices** 11](#_Toc205294708)

[**6.** **Comparison to models using routine HIV surveillance data only** 17](#_Toc205294709)

[**7.** **Estimation of pre- and post-migration** 20](#_Toc205294710)

# **Comparison of new HIV diagnoses over time in MSIS and NPR**

Including all patients in NPR first registered with an ICD-10 code for HIV (B20 – B24 or Z21) from 2008 to 2023 gave 6,736 individual patients. In the same period 3,662 new HIV diagnoses were notified to MSIS (Figure S1). The large discrepancy was mostly due to the high number of patients first registered with an HIV ICD-10 code in 2008 (2,181 in NPR, 299 in MSIS), reflecting the first year of data available from NPR in the dataset. I removed those first registered with an HIV ICD-10 code in 2008 – 2010, as much of the discrepancy between MSIS and NPR in these years likely reflected patients first diagnosed before 2008 whose first registration with B20 – B24 or Z21 in the study period reflected a consultation for clinical follow-up. From 2011 – 2023 I identified 3,721 patients in NPR, compared to 2,823 cases in MSIS. I further removed 574 NPR patients who only had one HIV ICD-10 code registered. Given high retention in care in Norway (treatment uptake 95% – 98%), these may be coding errors in NPR or patients with a diagnosed HIV infection who received a treatment refill while temporarily in Norway. The remaining discrepancy between MSIS and NPR from 2011 – 2023 was 324 individuals, ranging from 3 – 49 per year (Figure S2). Figure S3 shows the break down by country of birth. The remaining discrepancy may be due to first NPR consultations for some patients still not being reflective of first HIV diagnosis dates or some underreporting of new HIV diagnoses to MSIS. Nonetheless, the similarity in the number of ‘first registration with HIV’ in each data sources and comparability of the trend indicate that the NPR data are a good proxy for new HIV diagnoses, as notified to MSIS.

*
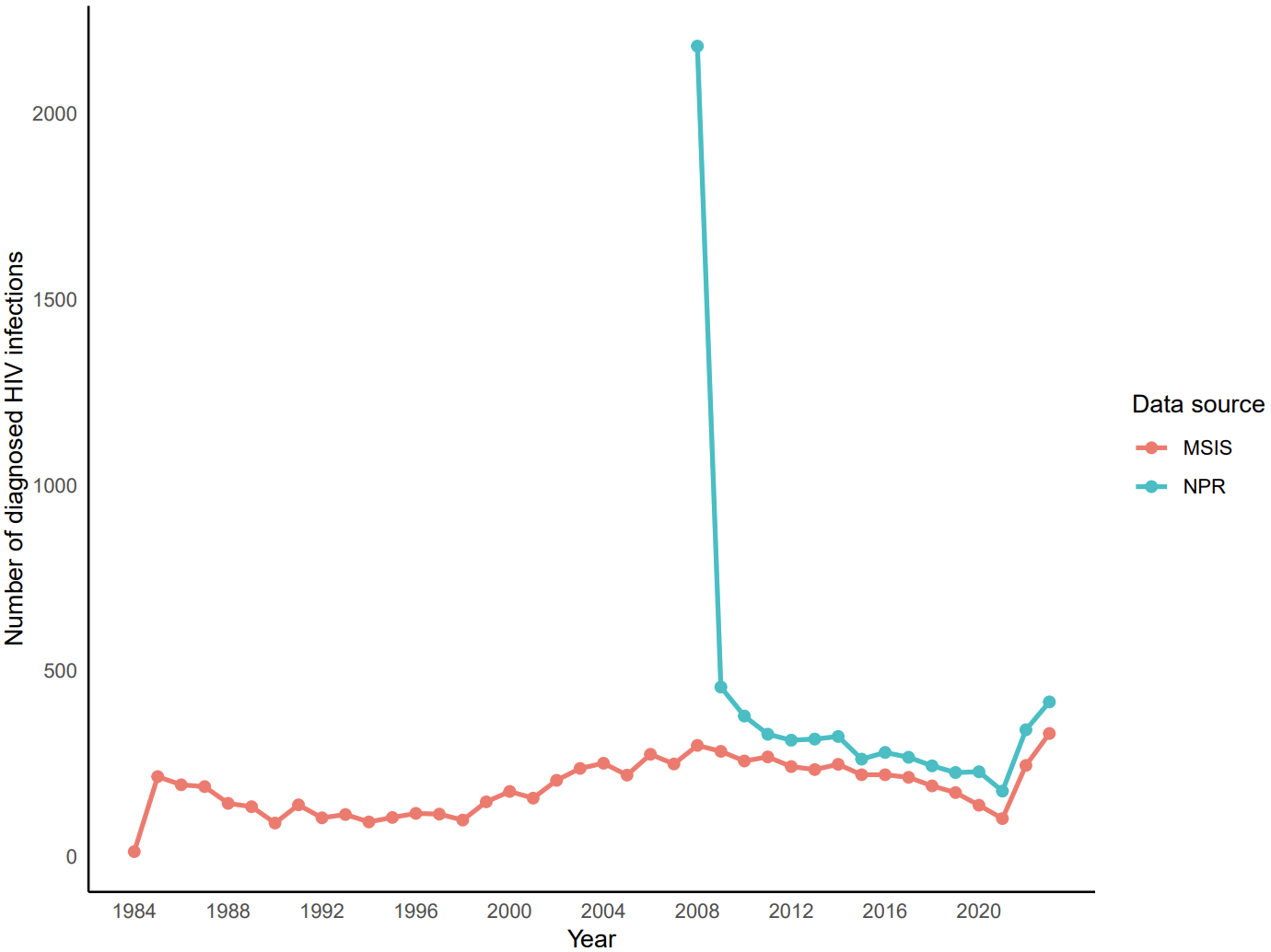
*

*Figure S1. Number of persons first registered with HIV in MSIS (1984 – 2023) or NPR (2008 – 2023), by data source and year.*


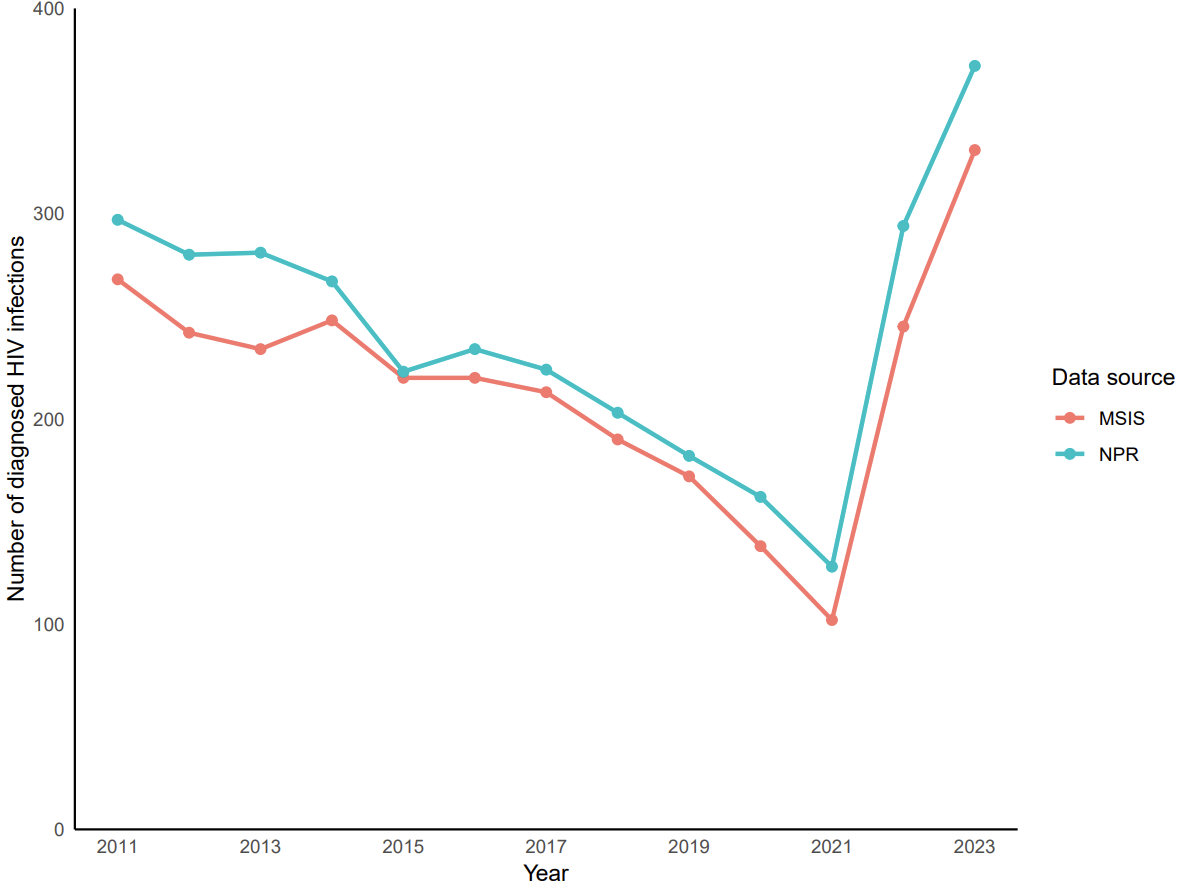


*Figure S2. Number of persons first registered with HIV, by data source and year, 2011 – 2023, excluding NPR patients who only* *had one HIV ICD-10 code (B20 – B24 or Z21) registered.*

*
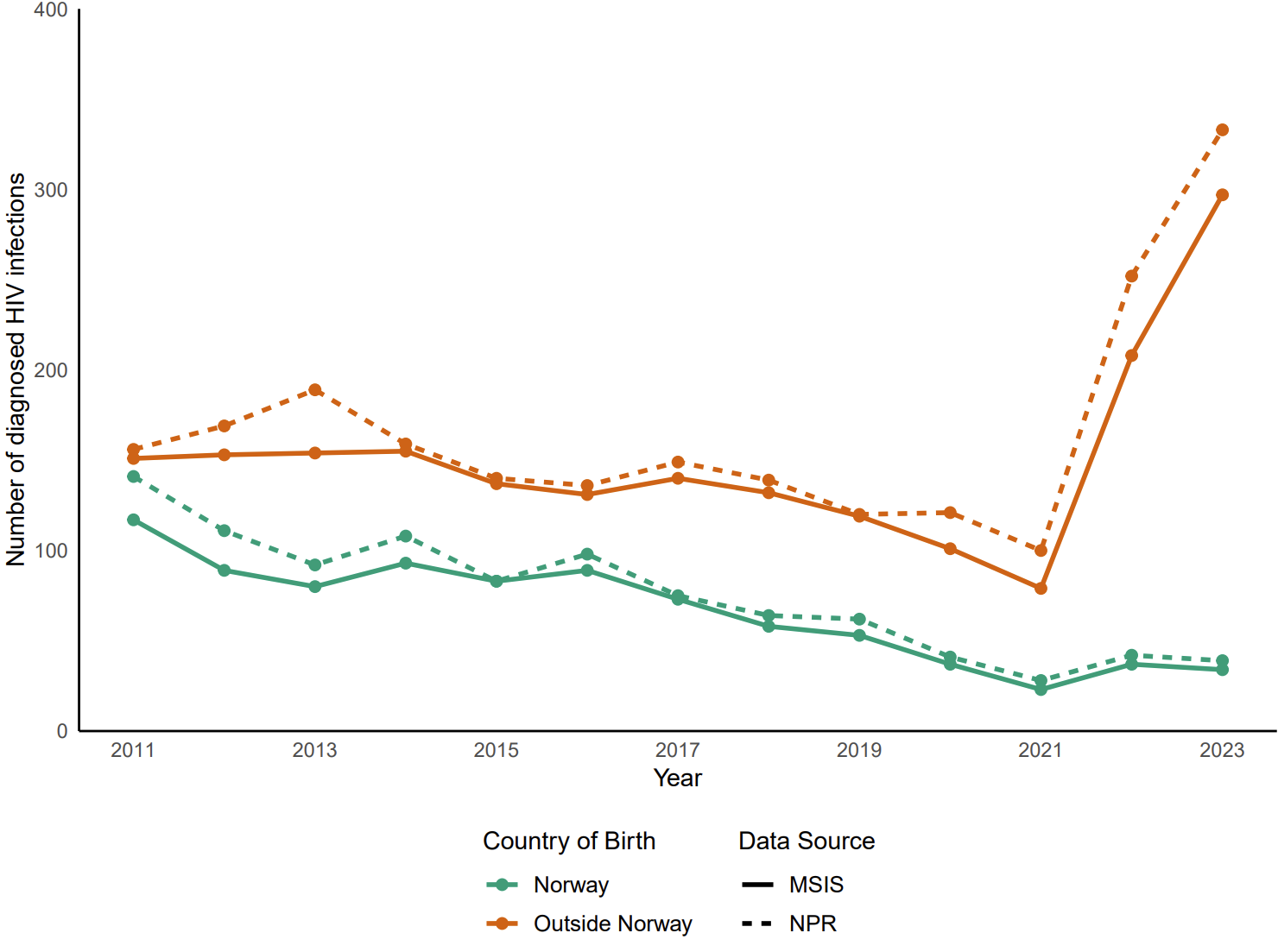
*

*Figure S3. Number of persons first registered with HIV, by data source, year and country of birth, 2011 – 2023, excluding NPR patients who only* *had one HIV ICD-10 code (B20 – B24 or Z21) registered.*

# **Defining AIDS based on NPR data and comparison to AIDS cases notified in MSIS**

In Norway, persons diagnosed with AIDS have been notified nominatively to MSIS since 1983. This notification has been done separately from HIV notifications. On HIV notifications, AIDS may be specified if the HIS and AIDS diagnoses were concurrent. A reported limitation of HIV surveillance in MSIS is that AIDS cases are underreported. When using the HIV modelling tool, incomplete reporting of AIDS diagnoses may affect estimating the incidence curve from the date of diagnosis.

To investigate the level of underreporting of AIDS diagnoses to MSIS and to ensure that more complete data on AIDS diagnoses were used in the modelling, I defined AIDS diagnoses using ICD-10 codes from NPR (Table S1). The codes selected were specific for AIDS-defining illnesses and cover most, but not all AIDS-defining illnesses (like nocardiosis or strongyloidiasis of internal organ). When HIV-specific codes were not available for AIDS-defining illnesses, I used non-HIV disease-specific codes. I also identified some non-HIV disease-specific codes for AIDS-defining illnesses that also had HIV-specific codes, to explore how the coding of HIV vs. non-HIV codes affected the results.

Figure S4 presents the trend over time. From 2007 a comparable number of AIDS diagnoses were identified in new AIDS and HIV notifications in MSIS. Among 69 HIV/AIDS notifications (i.e. HIV notifications with AIDS reported as diagnosed simultaneously) and 68 AIDS notifications from 2019 with a national identify number in MSIS, 62 overlapped. From 2019 – 2023 there were a total of 81 HIV/AIDS notifications and 90 AIDS notifications. This suggests that AIDS notifications to MSIS in recent years predominantly reflect AIDS diagnosed at the same time as HIV infection.

From 2011 – 2023, 320 additional AIDS diagnoses that could not be linked to an HIV/AIDS or AIDS notification in MSIS were identified in NPR ICD-10 codes, ranging from 13 – 47 per year. Of these 320, 179 (56%) had a HIV-specific code, 66 (21%) had a non-HIV, disease-specific code and 75 (23%) had both. B20.6 (n=57), B20.0 (n=45) and B20.4 (n=41) were the most common HIV-specific codes, followed by B22.2 (n=26), B22.0 (n=23), B20.2 (n=19), B21.2 (n=17) and B21.0 (n=13). All other codes had n<10. Among the 66 with only non-HIV disease specific codes, A15 (n=15), A16 (n=10), C53 (n=9), C58.2 (n=7) and C83 (n=7) were the most common. All other codes had n < 5.

Among these 320, the median time from HIV diagnosis (or first registration of an HIV code in NPR) to AIDS diagnosis was 1 month (IQR: 0 – 11 months). There were 107 born in Norway, ranging from 1 – 15 per year, with a median time to AIDS diagnosis of 0 months (IQR: 0 – 2 months). There were 196 born outside Norway, ranging from 7 – 25 per year, with a median time to AIDS diagnosis of 1 month (IQR: 0 – 16 months). There were 17 with unknown country of birth. Among the 34 who could be linked to an HIV case notified to MSIS from 2019 (first year with national identifiers in MSIS), the median time from HIV diagnosis to AIDS diagnosis was 1 month (IQR: 0 – 7 months).

There were 22 AIDS notifications that could not be linked to an AIDS case in NPR, as defined in Table S1. Of these 22, 6 were reportedly cases of Kaposi sarcoma. For the remaining 16, n was < 3 for other AIDS-defining conditions.

This suggests that there is notable underreporting of new AIDS diagnoses to MSIS, of which a notable proportion are diagnosed with AIDS around the time of HIV diagnosis.

*Table S1: ICD-10 codes used to define AIDS diagnoses in NPR.*

| **AIDS‐defining illness** | **HIV-specific code** | **Non-HIV, disease-specific code** |
| --- | --- | --- |
| Pulmonary tuberculosis, disease caused by M. tuberculosis, extrapulmonary, ay mycobacterial disease caused by bacteria other than M. tuberculosis, disseminated. | HIV disease resulting in mycobacterial infection, HIV disease resulting in tuberculosis (B20.0). | Respiratory tuberculosis, bacteriologically and histologically confirmed (A15), or not confirmed bacteriologically or histologically (A16), tuberculosis of nervous system (A17), other organs (A18) or miliary tuberculosis (A19), infection due to other mycobacteria (A31). |
| Salmonella (non-typhoid) septicaemia, recurrent | – | Salmonella sepsis (A02.1) at least twice in a 12-month period, at least 3 months between episodes. |
| Cytomegalovirus retinitis with loss of vision | HIV disease resulting in cytomegaloviral disease (B20.2) | – |
| Recurrent bacterial pneumonia | – | Bacterial pneumonia (J13 – J15) at least twice in a 12-month period, at least 3 months between episodes. |
| Candidiasis of the oesophagus or lung. | HIV disease resulting in candidiasis (B20.4) | – |
| Coccidioidomycosis, disseminated (at a site other than or in addition to lungs or cervical or hilar lymph nodes) | – | Cutaneous coccidioidomycosis (B38.3)  Coccidioidomycosis meningitis (B38.4)  Disseminated coccidioidomycosis (B38.7) |
| Histoplasmosis, disseminated (other than or in addition to lungs or cervical or hilar lymph nodes) | – | Disseminated histoplasmosis capsulati, Generalized histoplasmosis capsulati (B39.3) |
| Pneumocystis carinii pneumonia | HIV disease resulting in Pneumocystis jirovecii pneumonia, HIV disease resulting in Pneumocystis carinii pneumonia (B20.6) | Pneumocystosis (B48.5) |
| Isosporiasis with diarrhoea persisting >1 month | – | Isosporiasis (A07.3) |
| Toxoplasmosis of the brain in patient >1 month of age | – | Toxoplasma meningoencephalitis (B58.2) |
| Kaposi sarcoma at any age | HIV disease resulting in Kaposi sarcoma (B21.0) | Kaposi sarcoma (C46) |
| Non-Hodgkin’s lymphoma | Burkitt’s lymphoma (B21.1)  HIV disease resulting in other types of non-Hodgkin lymphoma (B21.2) | Burkitt lymphoma (C83.7)  Follicular lymphoma (C82), non-follicular lymphoma (C83), mature T/NK-cell lymphomas (C84), other and unspecified non-Hodgkin lymphoma (C85), other specified T/NK-cell lymphoma (C86) |
| Cervical cancer, invasive | – | Malignant neoplasm of cervix uteri (C53) |
| HIV encephalopathy | HIV disease resulting in encephalopathy, HIV dementia (B22.0) | – |
| HIV wasting syndrome | HIV disease resulting in wasting syndrome, HIV disease resulting in failure to thrive, Slim disease (B22.2) | – |

*
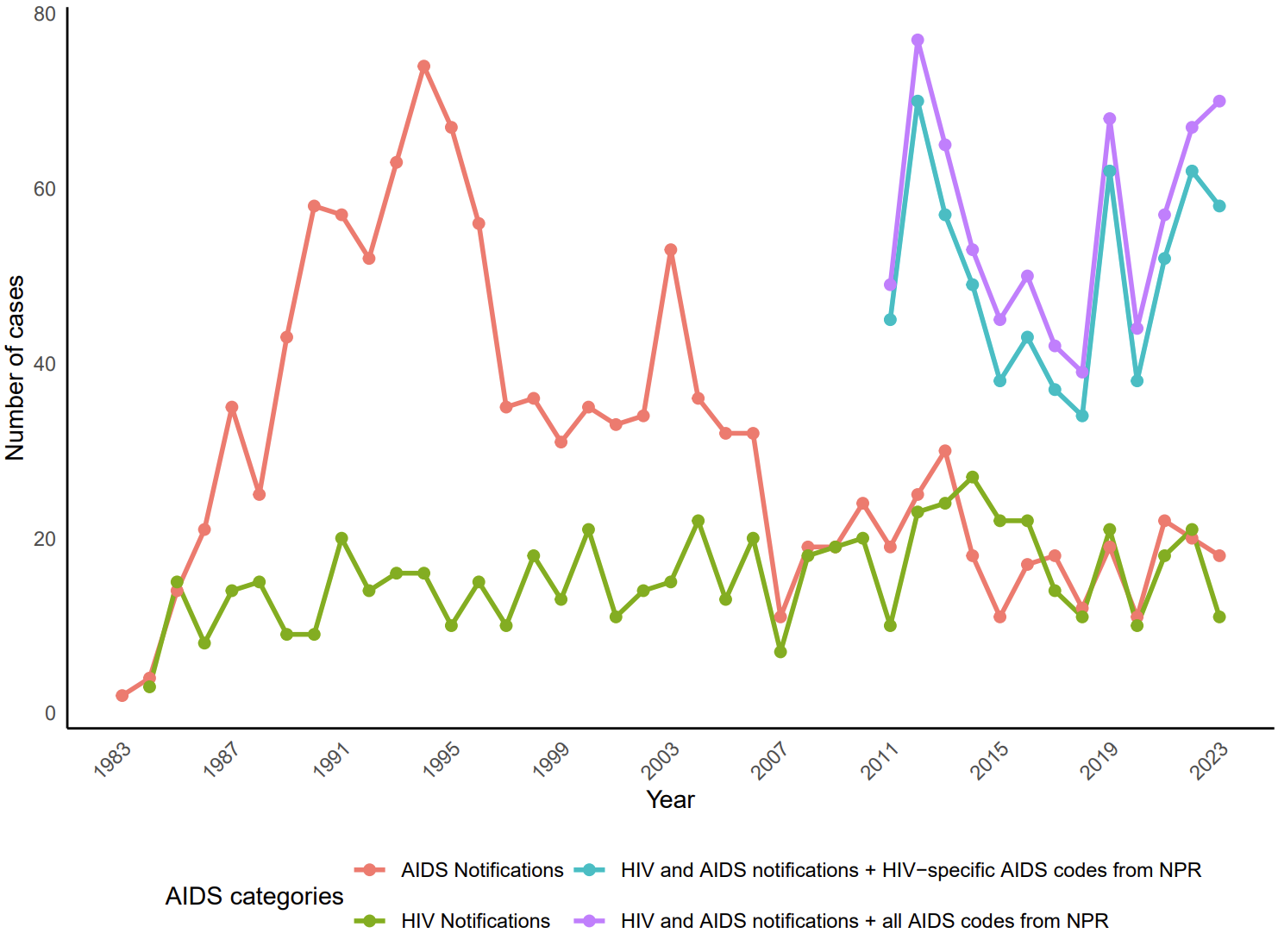
*

*Figure S4. Number of persons registered with AIDS in HIV notifications to MSIS, AIDS notifications to MSIS or with an ICD-10 code used to define AIDS diagnoses in NPR (Table S1), by year, 1983 – 2023. NPR data are only included from 2011, as this is what was used in the modelling.*

# **Additional details on model inputs and parameters**

The model input data included the age in years, sex, mode of transmission, country of birth, HIV diagnosis date, AIDS diagnosis date, CD4 count at diagnosis, date of first CD4 count (same as date of HIV diagnosis), and the year of death or outmigration. For the linked data, the migration date was also included. As the input data were extracted from September 2024 onwards, I assumed no reporting delay. The tool can perform multiple imputations for missing values of age, country of birth, CD4 count and mode of transmission. However, as the completeness of data on these variables by year was close to 100% or 0% (Figure S5), I did not run any imputations.

For the ‘Region for Migration Module Parameter’, the countries of birth were categorised into regions using the model preset ‘REPCOUNTRY + UNK + EASTERN EUROPE + EUROPE-NORTH AMERICA-OTHER + AFRICA + ASIA + OTHER’.

Four knots were used for the incidence curve. I assumed that the curve started at zero, and selected options for not using data from the start of the epidemic and preventing sudden changes at the end of the observation period. For years without CD4 data, an extra diagnosis rate due to non-AIDS symptoms was not specified. I used a negative binomial for the maximum likelihood distribution.

*
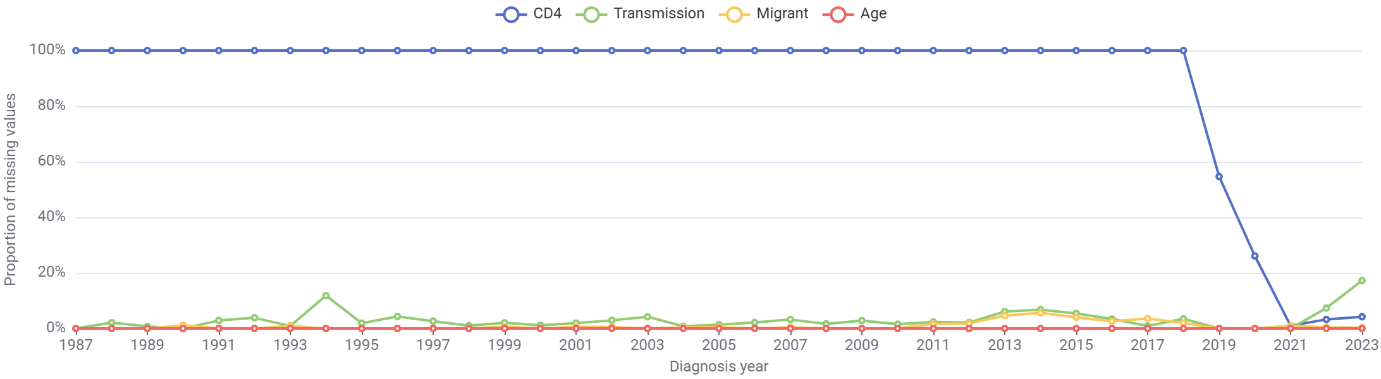
*

*
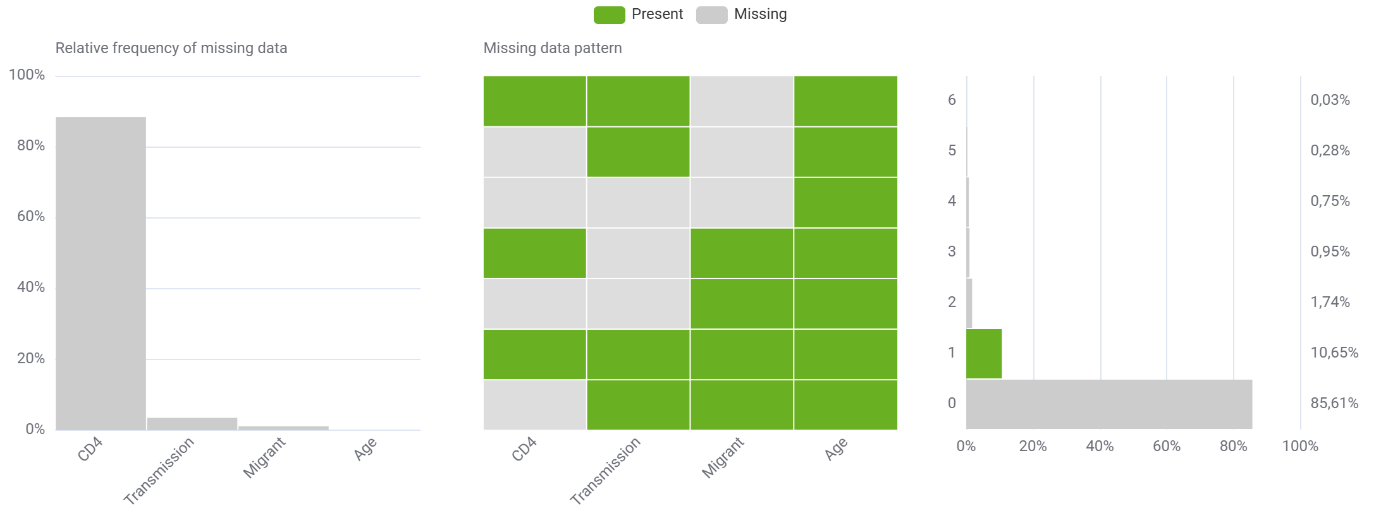
*

*Figure S5. Data completeness for age, mode of transmission, CD4 count at diagnosis and country of birth (‘migrant’).*

# **Diagnosis matrix and goodness-of-fit**

The tool requires that a diagnosis matrix is defined to determine the shape of the diagnosis probability. I tested several different diagnosis matrices to find the best goodness of fit. If the goodness of fit was similar, I chose the simpler matrix. The diagnosis matrix used for most models included four time periods: 1980 – 1983, 1984 – 2018 (including jump and change during period) 2019 – 2021 (including change by CD4 count and during period) and 2022 – 2023 (including change by CD4 count and during period) (Figure S6). The periods 2019 – 2021 and 2022 – 2023 were chosen to ensure a good fit to the trend in new HIV diagnoses at the end of the study period, driven by notable changes in migration patterns due to the COVID-19 pandemic and outbreak of the war in Ukraine. Also, CD4 count data were first available in MSIS in 2019. The goodness-of-fit statistic for the main model was 313. The fit to HIV diagnoses and HIV/AIDS diagnoses is presented in the Figures S7 – S8.

The diagnosis matrix used for each other population modelled was the same as in Figure S6, except that additional periods were added for heterosexual transmission from 1999 – 2019, and for MSM from 2002 – 2010 and 2011 – 2019, as these gave a better fit to data in the early part of the epidemic. Model fits to some other modelled populations and diagnosis matrices are presented in part 5 of this supplement. Model fits for all modelled populations are available on request.

*
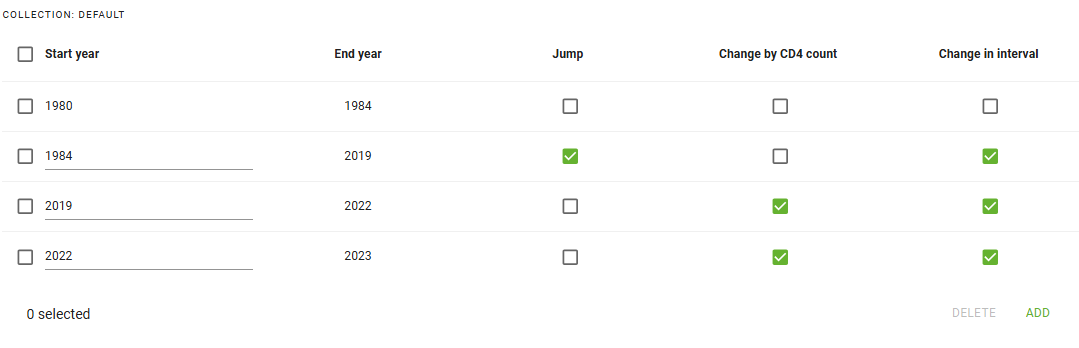
*

*Figure S6. Diagnosis matrix used in main modelling.*

*
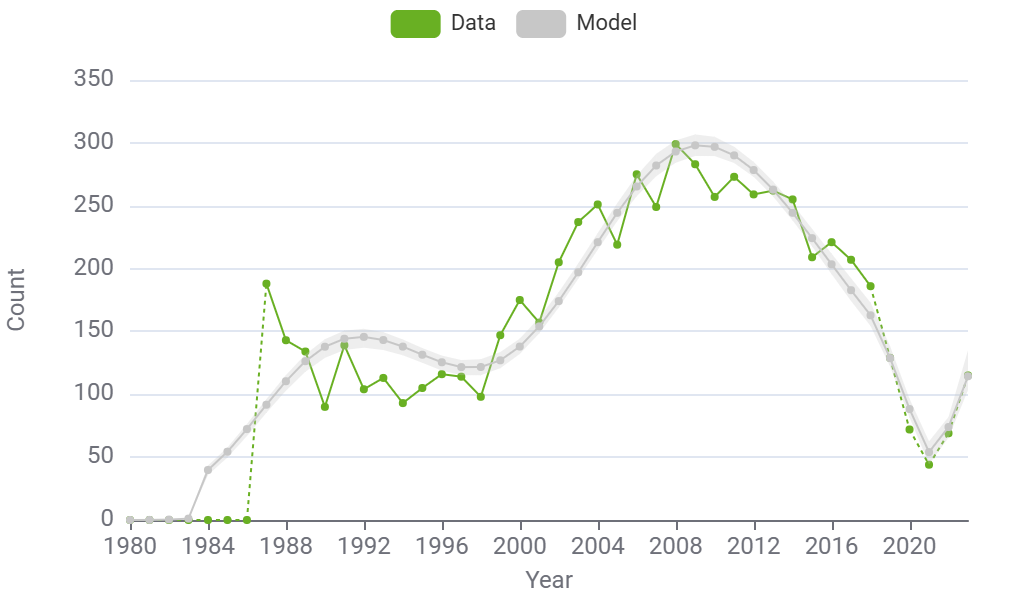
*

*Figure S7. Model fit to HIV diagnoses*

*
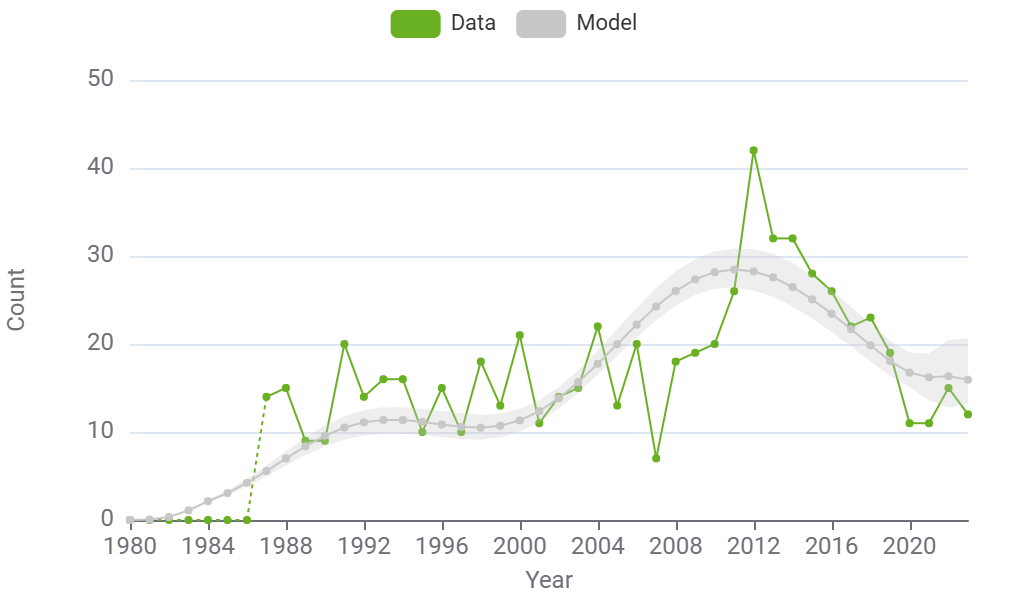
*

*Figure S8. Model fit to HIV/AIDS diagnoses*

# **Alternative diagnosis matrices**

To explore the sensitivity of the main model to the choice of diagnosis matrix (see part 4), alternatives were tested. Results are presented in Table S2 and the goodness of fit in Figures S9 – S18.

I tested adding a period at 2011 (when the linked data were first used in the modelling and around the time when some low threshold testing services for some key populations were first implemented, like Checkpoint in Oslo in 2012) and 2015 (the first year when it was specified that refugees and asylum seekers from high-prevalence countries should be offered testing ‘within three months of arrival’). Adding these two periods gave wider confidence intervals, slightly higher incidence and a lower number undiagnosed. Similar results were observed for migrants.

Having one time period from 2011 onwards, i.e. ignoring the changing trend in the new HIV diagnoses at the end of the study period, resulted in higher incidence and lower number undiagnosed, but a poorer model fit. However, for all Norwegian-born cases and Norwegian-Born MSM, this matrix gave a similar fit to data and more balanced confidence intervals. Results were similar, with a slightly lower number undiagnosed and a higher diagnosed fraction. Using this diagnosis matrix for Norwegian-born MSM, 95% diagnosed was first exceeded in 2018, instead of 2020 in the main model. Using this diagnosis matrix did not notably change results for Norwegian-Born heterosexuals.

*Table S2. Model estimates for 2023 using alternative diagnosis matrices.*

| **Population modelled and diagnosis matrix** | **Number of new HIV infections (95% CI)** | **Number of PLHIV (95% CI)** | **Number of undiagnosed infections (95% CI)** | **Proportion undiagnosed (95% CI)** |
| --- | --- | --- | --- | --- |
| **All PLHIV** | | | | |
| Main model | 13 (11 – 50) | 6 726 (6 683 – 6 856) | 234 (207 – 336) | 96.5 (95.1 – 96.9) |
| Period added at 2011 and 2015 | 38 (11 – 93) | 6 714 (6 616 – 6 920) | 224 (142 – 389) | 96.7 (94.4 – 97.9) |
| One period from 2011 onwards | 58 (41 – 80) | 6 653 (6 581 – 6 715) | 168 (131 – 217) | 97.5 (96.8 – 98.0) |
| **Norwegian-born** | | | | |
| Main model | 5 (3 – 67) | 2 645 (2 549 – 2 844) | 109 (75 – 289) | 95.9 (89.9 – 97.1) |
| One period from 2011 onwards | 18 (4 – 35) | 2 615 (2 530 – 2 694) | 83 (53 – 153) | 96.8 (94.2 – 97.9) |
| **Migrants** | | | | |
| Main model | 9 (8 – 36) | 4 098 (4 030 – 4 189) | 128 (103 – 183) | 96.9 (95.6 – 97.4) |
| Period added at 2011 and 2015 | 30 (7 – 56) | 4 080 (4 007 – 4 167) | 119 (62 – 194) | 97.1 (95.3 – 98.5) |
| **Norwegian-born MSM** | | | | |
| Main model | 7 (1 – 30) | 1 357 (1 294 – 1 434) | 47 (17 – 110) | 96.6 (92.3 – 98.7) |
| One period from 2011 onwards | 7 (1 – 16) | 1 336 (1 270 – 1 406) | 26 (13 – 50) | 98.1 (96.3 – 99.0) |
| **Norwegian-born heterosexual transmission** | | | | |
| Main model | 3 (2 – 24) | 937 (904 – 1 040) | 69 (50 – 132) | 92.6 (87.1 – 94.5) |
| One period from 2011 onwards | 4 (2 – 28) | 942 (853 – 1 055) | 69 (31 – 163) | 92.6 (84.5 – 96.5) |

*
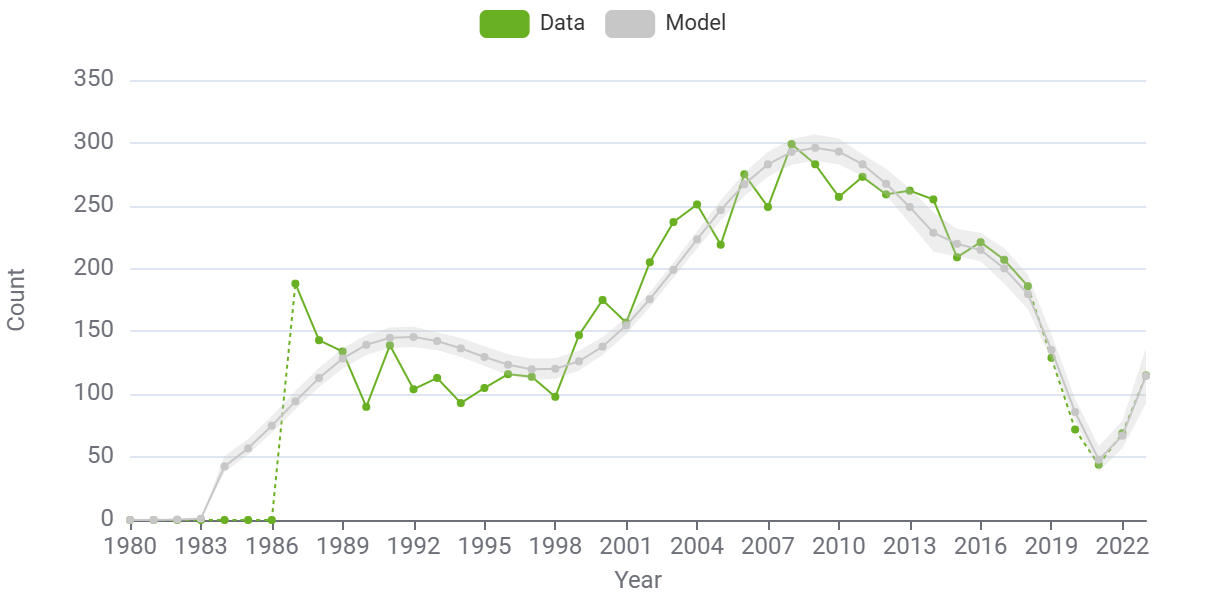
*

*Figure S9. Model fit to HIV diagnoses, all PLHIV, period added at 2011 and 2015.*

*
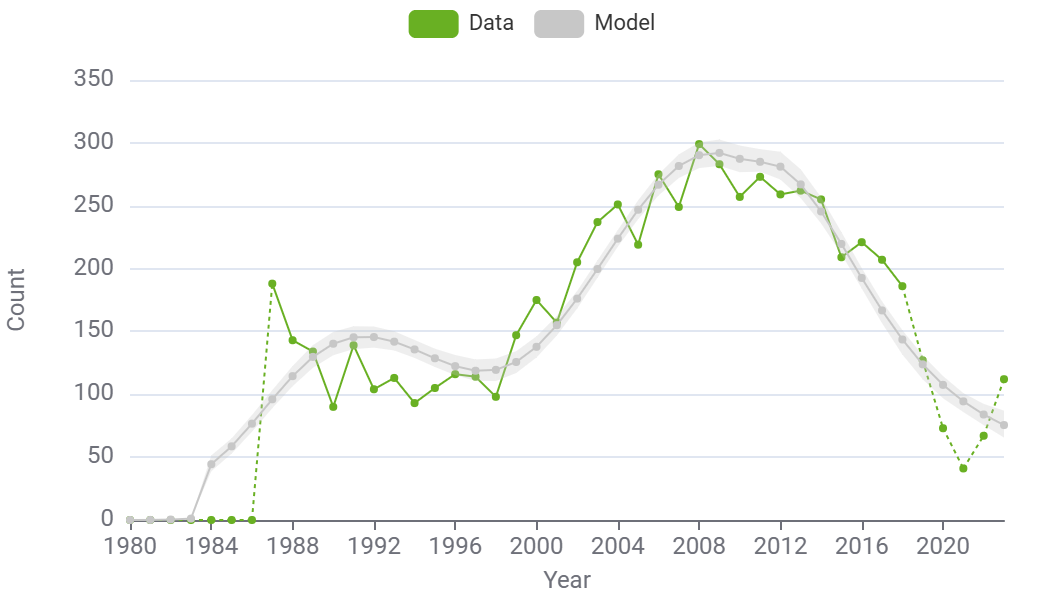
*

*Figure S10. Model fit to HIV diagnoses, all PLHIV, one period from 2011 onwards.*

*
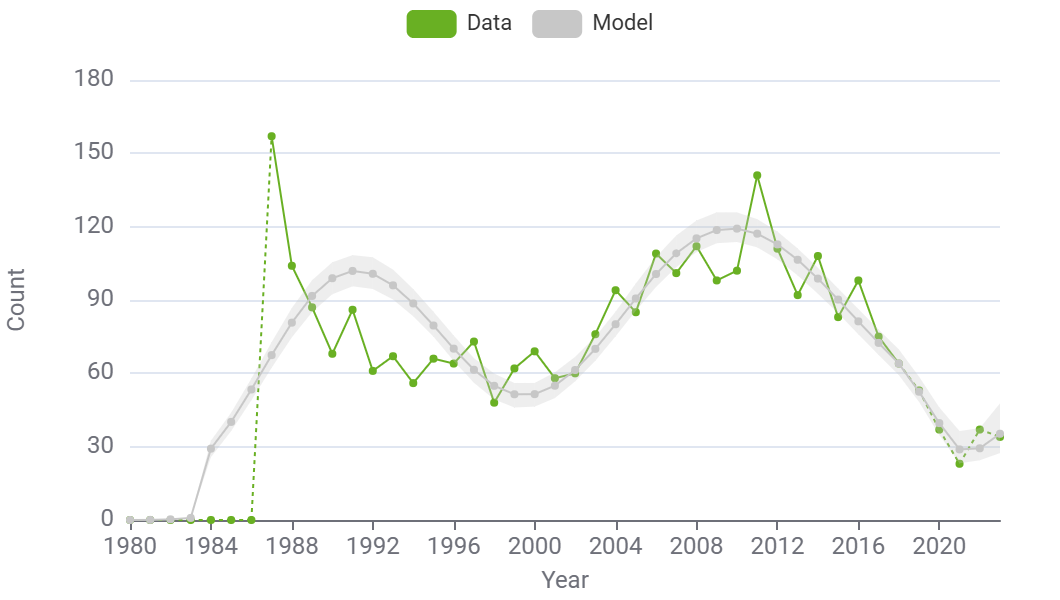
*

*Figure S11. Model fit to HIV diagnoses, Norwegian-born, main model.*

*
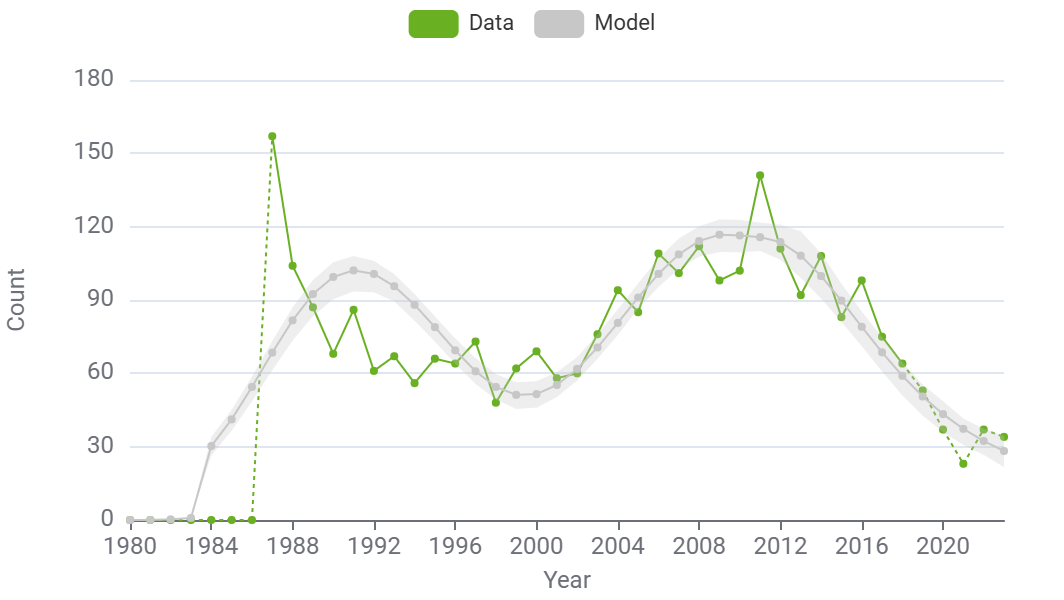
*

*Figure S12. Model fit to HIV diagnoses, Norwegian-born, one period from 2011 onwards.*

*
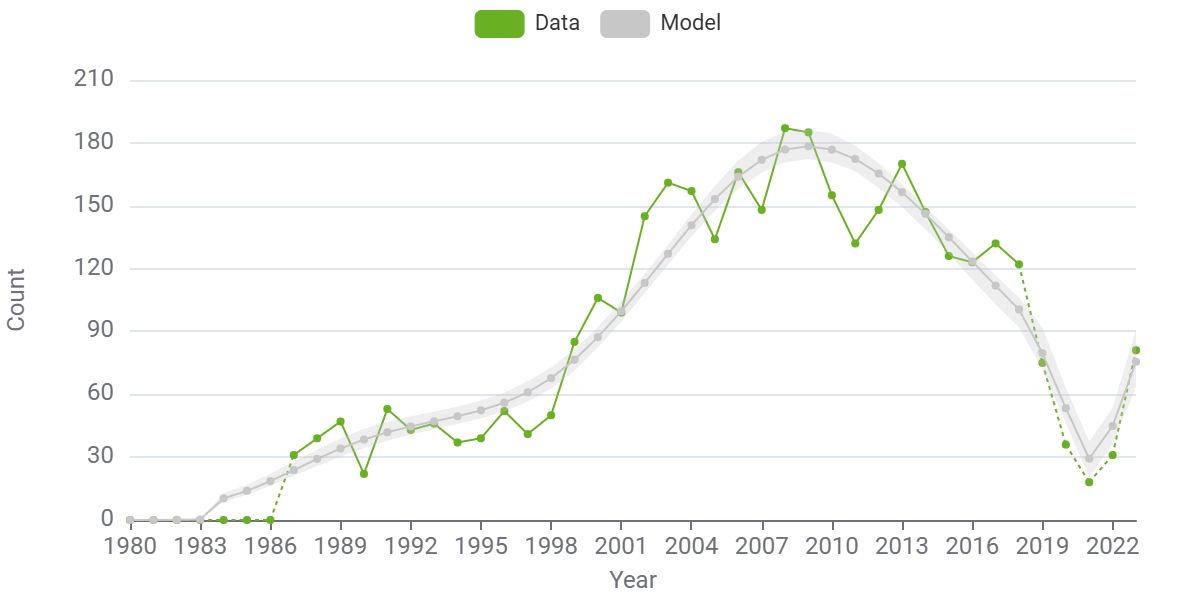
*

*Figure S13. Model fit to HIV diagnoses, migrants, main model.*

*
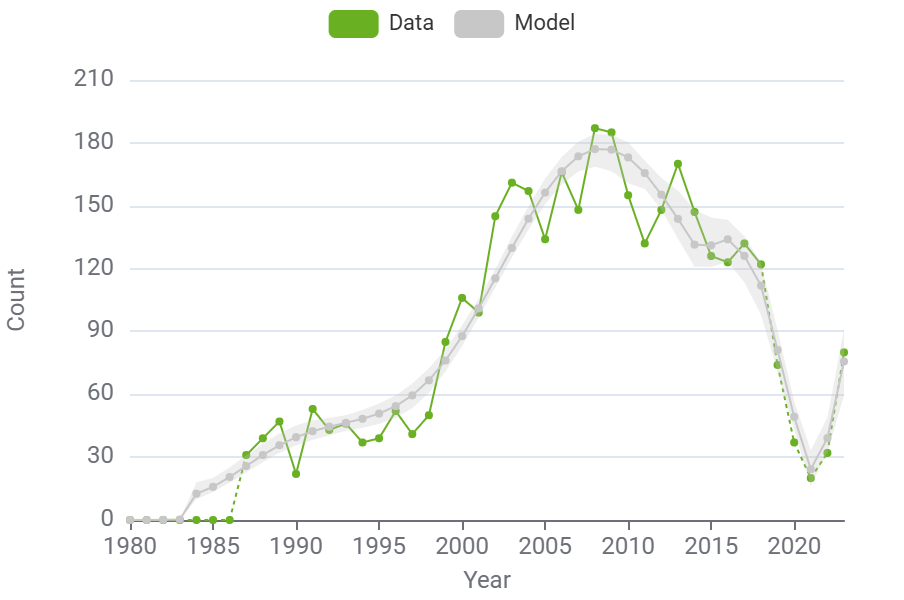
*

*Figure S14. Model fit to HIV diagnoses, migrants, period added at 2011 and 2015.*

*
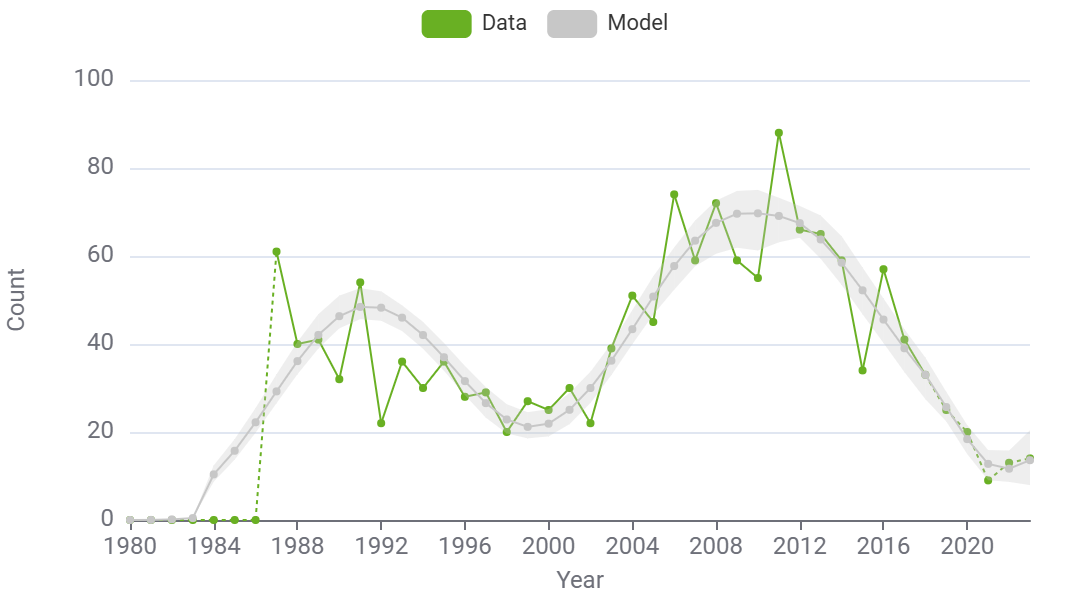
*

*Figure S15. Model fit to HIV diagnoses, Norwegian-born MSM, main model.*

*
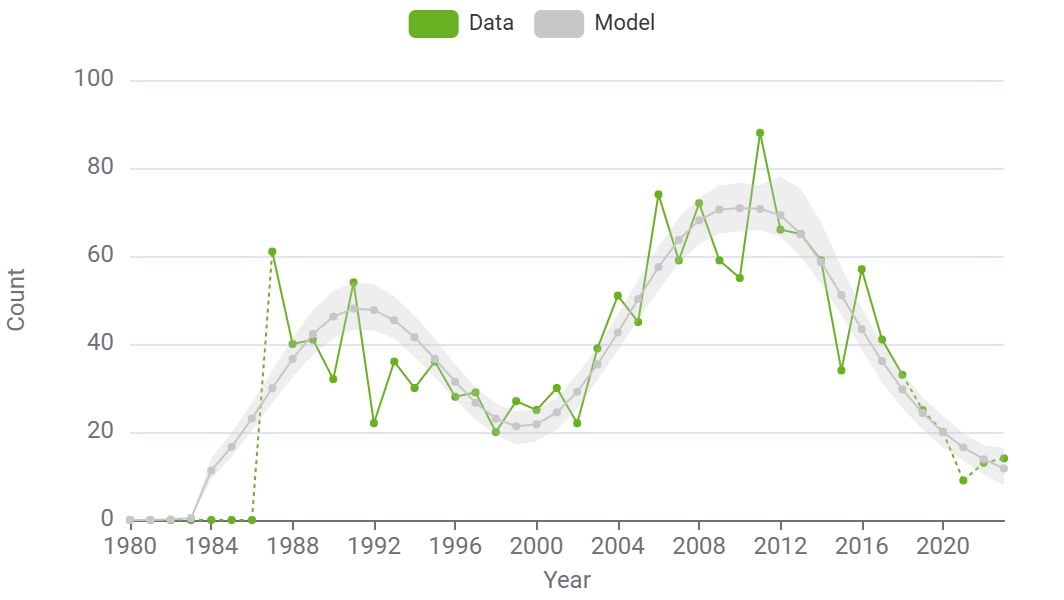
*

*Figure S16. Model fit to HIV diagnoses, Norwegian-born MSM, one period from 2011 onwards.*

*
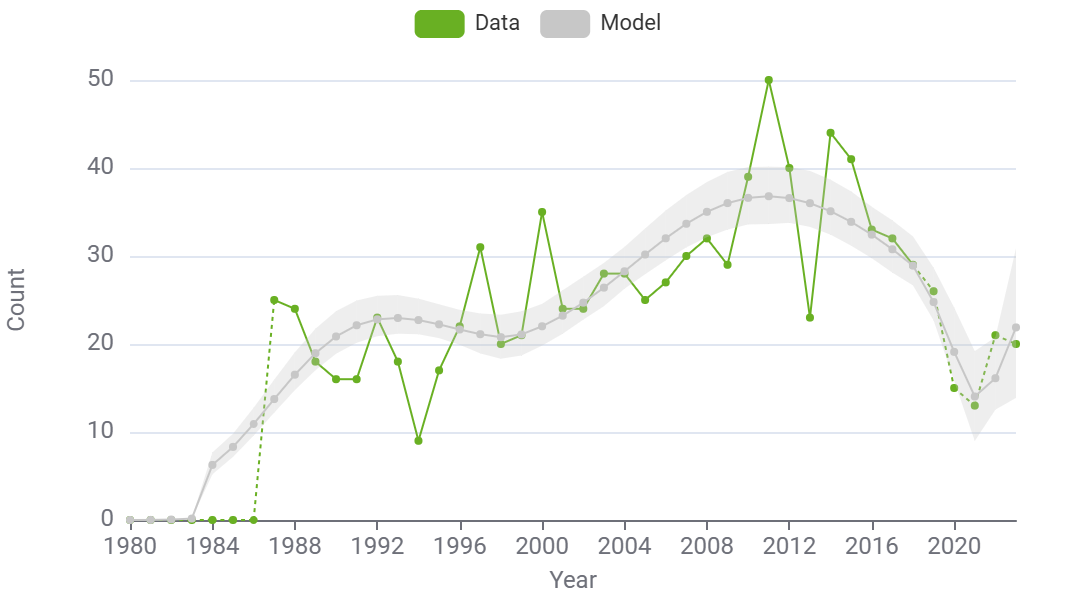
*

*Figure S17. Model fit to HIV diagnoses, Norwegian-born heterosexual transmission, main model.*

*
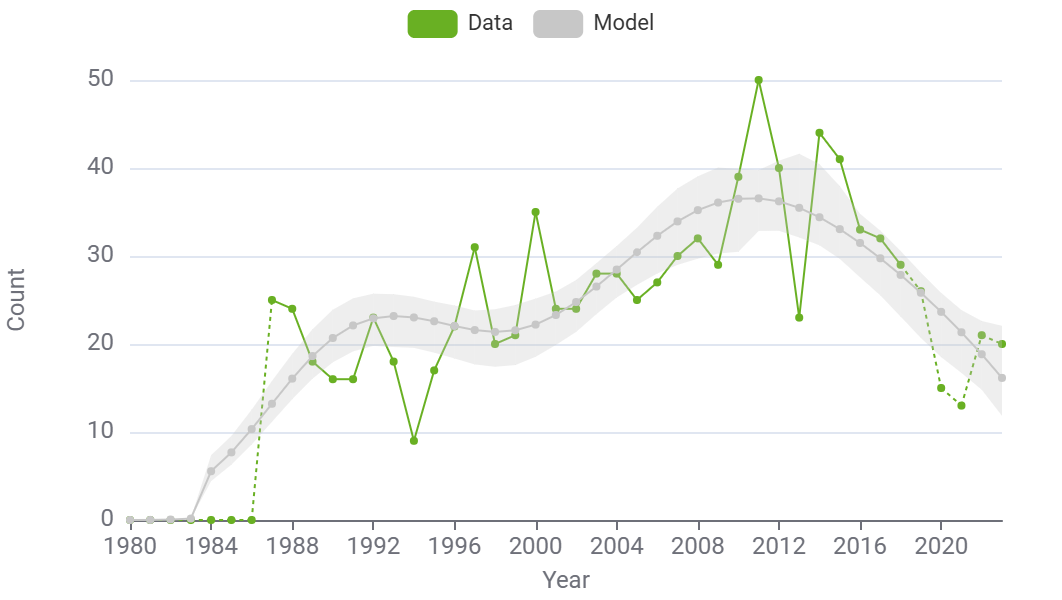
*

*Figure S18. Model fit to HIV diagnoses, Norwegian-born heterosexual transmission, one period from 2011 onwards.*

# **Comparison to models using routine HIV surveillance data only**

To explore how the availability of data beyond routine HIV surveillance data through registry linkage affected the results, I also ran models only utilising routine HIV surveillance data from MSIS. This dataset included MSIS data from 1987 – 2023 and was not linked to data from other registries. Pre-migration infections, underreporting of AIDS diagnoses and underreporting of deaths and out-migrations could not be taken into account. The model fit to the routine data is presented in Figures S19 and S20. Results compared to the main model are presented in Table S3 and Figure S21.

Using the routine data only for all PLHIV and for migrants increased the estimates of incidence, the number undiagnosed and the number of PLHIV in 2023. The estimated diagnosed fraction decreased. This highlights the impact of not taking pre-migration infection into account when modelling the HIV epidemic in Norway. I also ran an additional model on all PLHIV excluding those diagnosed with HIV before arrival to Norway, which will exclude some pre-migration infections from incidence estimations. This gave an estimated incidence in 2023 of 152 (95% CI: 77 – 250) new infections, with 591 (95% CI: 548 – 632) undiagnosed infections. The slightly higher number of PLHIV estimated using the routine data, closely reflects the difference in the number of deaths and out-migrations between the datasets.

Using the routine data on Norwegian-born persons gave higher estimates of incidence and the number undiagnosed, and a lower diagnosed fraction in 2023, with some overlap between point estimates and confidence intervals. This suggests that the approximate three- to four-fold underreporting of concurrent HIV/AIDS diagnoses (see part 2) impacted the estimates. This may be a consequence of data availability in Norway, where CD4 count was first reported in 2019. More complete data on HIV/AIDS diagnoses in the linked data from 2011 – 2023, i.e. before and after CD4 count data were first reported may better nuance the CD4 count distribution, providing a more stable trend in ‘late’ diagnoses, and subsequent back-calculation to determine new and undiagnosed infections.

*
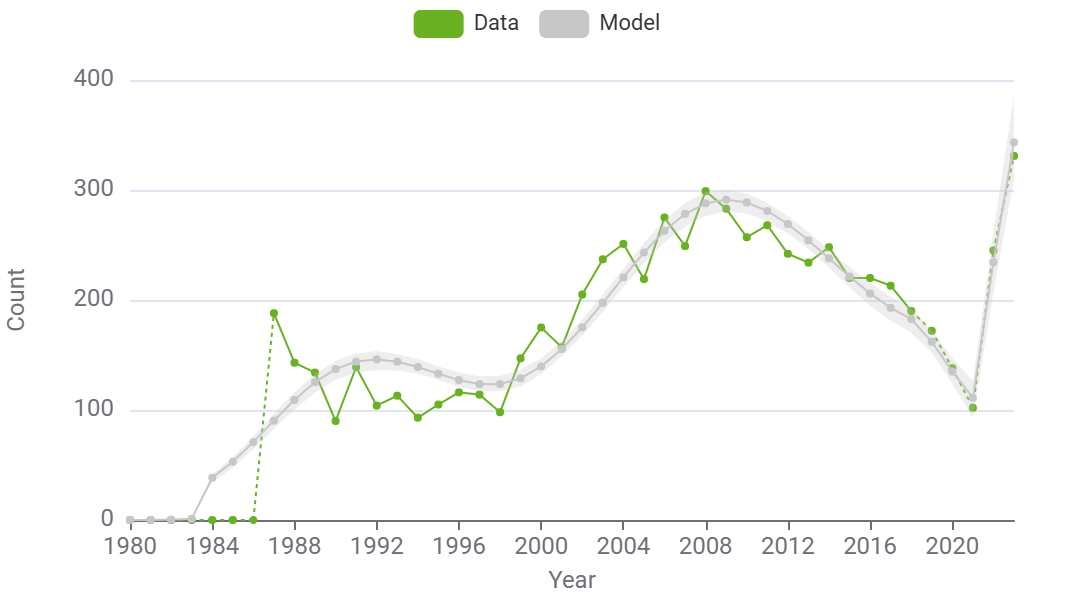
*

*Figure S19. Model fit to HIV diagnoses, routine surveillance data only*

*
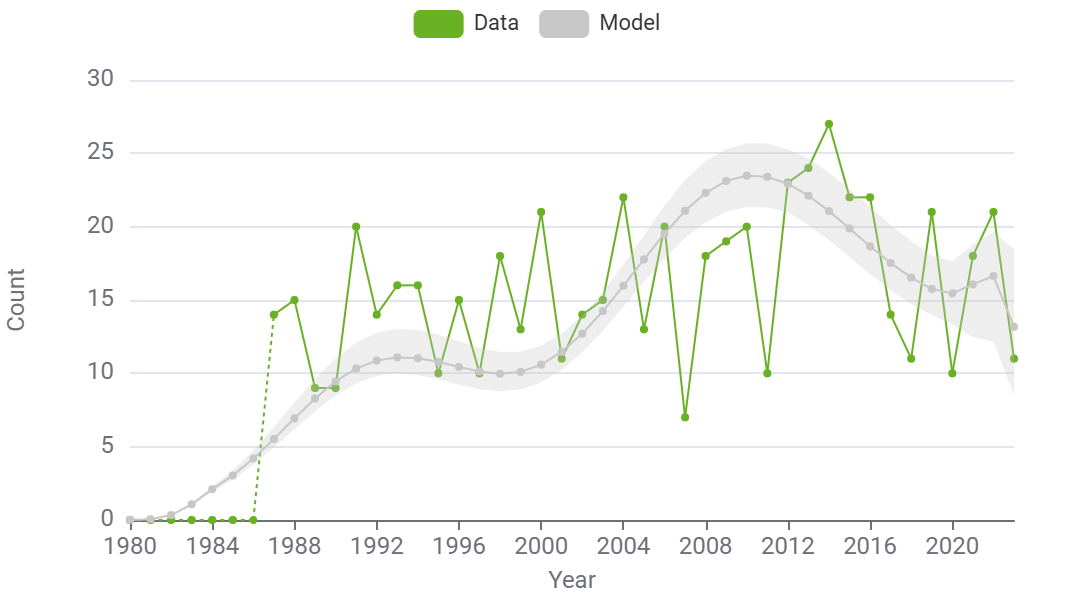
*

*Figure S20. Model fit to HIV/AIDS diagnoses, routine surveillance data only*

*Table S3. Key model outputs by input data and population modelled*

| **Population and dataset** | **Number of diagnosed cases of HIV in model input data** | **Model outputs** | | | | |
| --- | --- | --- | --- | --- | --- | --- |
|  |  | **Number of new HIV infections in 2010 (95%CI)** | **Number of new HIV infections in 2023 (95%CI)** | **Number of PLHIV in 2023 (95%CI)** | **Number of undiagnosed PLHIV in 2023 (95%CI)** | **Proportion diagnosed in 2023 (95%CI)** |
| **All PLHIV** | | | | | | |
| Main model input data | 7,185 | 287 (281 – 295) | 13 (11 – 50) | 6 726 (6 683 – 6 856) | 234 (207 – 336) | 96.5 (95.1 – 96.9) |
| Routine surveillance data only | 7,014 | 274 (265 – 282) | 196 (152 – 251) | 6 939 (6 827 – 7 107) | 347 (258 – 524) | 95.0 (92.6 – 96.2) |
| **Norwegian-born** | | | | | | |
| Main model input data | 2,919 | 119 (111 – 125) | 5 (3 – 67) | 2 645 (2 549 – 2 844) | 109 (75 – 289) | 95.9 (89.9 – 97.1) |
| Routine surveillance data only | 2,829 | 107 (99 – 114) | 54 (3 – 109) | 2 795 (2 603 – 2 943) | 225 (50 – 384) | 91.9 (86.8 – 98.1) |
| **Migrants** | | | | | | |
| Main model input data | 4,266 | 170 (163 – 176) | 9 (8 – 36) | 4 098 (4 030 – 4 189) | 128 (103 – 183) | 96.9 (95.6 – 97.4) |
| Routine surveillance data only | 4,185 | 166 (158 – 172) | 190 (161 – 221) | 4 293 (4 223 – 4 398) | 264 (204 – 359) | 93.8 (91.8 – 95.2) |

CI: Confidence interval. PLHIV: People who live with HIV.


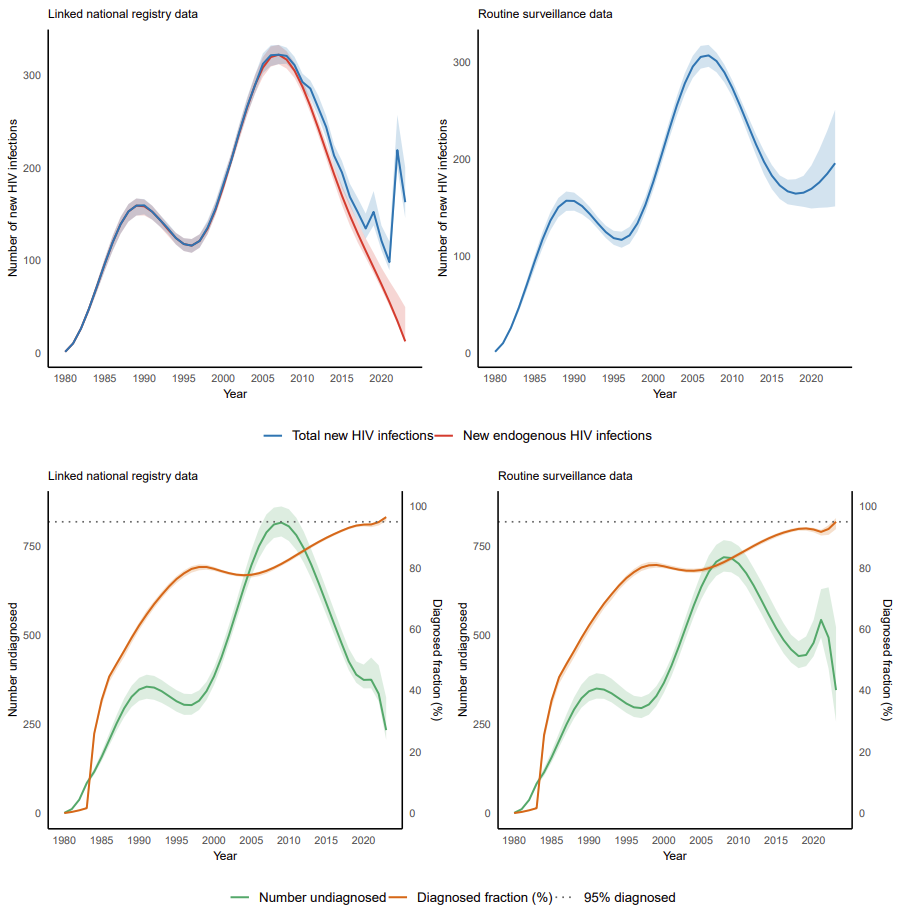


*Figure S21. Estimated number of total new HIV infections, endogenous infections, number of undiagnosed infections and the diagnosed fraction for different model input data, Norway, 1980 – 2023.*

Endogenous infections: infections among persons born or resident in Norway. The dotted line for 95% diagnosed indicates the UNAIDS goal of 95% of people living with HIV being diagnosed by 2025. The shaded area around the estimates line reflects the 95% confidence interval. The ‘Routine surveillance data’ dataset includes routine HIV surveillance data from MSIS from 1987 – 2023 only. For a description of the ‘Linked national registry data’ dataset, see the methods.

# **Estimation of pre- and post-migration**

The estimation of pre- or post-migration was included in all modelling using the main model input data from different national registries. Results from the estimation process are presented in Table S4, Table S5 and Figure S22. For further details on the method behind the estimation, see the tool manual at <https://www.ecdc.europa.eu/en/publications-data/hiv-platform-tool>.

The tool requires data on mode of transmission for the migration adjustment, but this is not available in NPR, which defined HIV diagnoses from 2011 – 2018 in the linked data. As the number of new diagnoses by year compared to MSIS was similar (see the supplement, part 1) I applied the distribution of mode of transmission in MSIS by year and region of origin (as categorised in the tool manual) to the NPR cases.

*Table S4. Selection of cases included in the estimation of pre- or post-migration*

| ***Priority*** | ***Missing variable*** | ***Number of excluded cases*** |
| --- | --- | --- |
| *1* | *Migrant region of origin is missing* | *76* |
| *2* | *Not considered a migrant, because region of origin is the reporting country* | *2,919* |
| *3* | *Migrant region of origin is not one of "AFRICA", "EUROPE-NORTH AMERICA", "ASIA", "OTHER"* | *0* |
| *4* | *Sex is not either "F" or "M"* | *0* |
| *5* | *Transmission is missing* | *138* |
| *6* | *Transmission is not of mode "MSM", "IDU", "HETERO" or "TRANSFU"* | *0* |
| *7* | *Date of arrival is before date of birth* | *1* |
| *8* | *Age is missing* | *0* |
| *9* | *Age is below 16* | *110* |
| *10* | *Number of years from risk onset to HIV diagnosis is missing* | *0* |
| *11* | *Date of HIV diagnosis is before risk onset* | *0* |
| *12* | *Date of arrival is missing* | *2,195* |
|  | ***Total excluded*** | ***5,439*** |
|  | ***Total used in estimation*** | ***1,746*** |

*Table S5. Results of the pre- or post-migration estimation by sex, age, mode of transmission and place of birth*

| **Category** | **Count** | **Pre-migration infection** | | **Post-migration infection** | |
| --- | --- | --- | --- | --- | --- |
|  |  | **Proportion** | **95% CI** | **Proportion** | **95% CI** |
| **Total** | 791 | 86% | 83% – 88% | 14% | 12% – 17% |
| **Sex** |  |  |  |  |  |
| Male | 425 | 82% | 77% – 85% | 18% | 15% – 23% |
| Female | 366 | 90% | 86% – 93% | 10% | 7% – 14% |
| **Age group** |  |  |  |  |  |
| < 25 years | 45 | 90% | 76% – 97% | 10% | 3% – 24% |
| 25 – 39 years | 395 | 89% | 85% – 92% | 11% | 8% – 15% |
| 40 – 54 years | 277 | 86% | 81% – 90% | 14% | 10% – 19% |
| ≥ 55 years | 74 | 63% | 51% – 74% | 37% | 26% – 49% |
| **Mode of transmission** |  |  |  |  |  |
| Heterosexual | 472 | 87% | 83% – 90% | 13% | 10% – 17% |
| MSM | 228 | 78% | 71% – 83% | 22% | 17% – 29% |
| Injecting drug use | 69 | 100% | 0% – 100% | 0% | 0% – 100% |
| Other | 22 | 95% | 73% – 99% | 5% | 1% – 27% |
| **Place of birth** |  |  |  |  |  |
| Africa | 250 | 86% | 80% – 90% | 14% | 10% – 20% |
| Asia | 101 | 75% | 64% – 83% | 25% | 17% – 36% |
| Eastern Europe | 241 | 97% | 93% – 99% | 3% | 1% – 7% |
| Europe (not Eastern) or North America | 142 | 76% | 68% – 83% | 24% | 17% – 32% |
| Other | 57 | 81% | 68% – 90% | 19% | 10% – 32% |

*
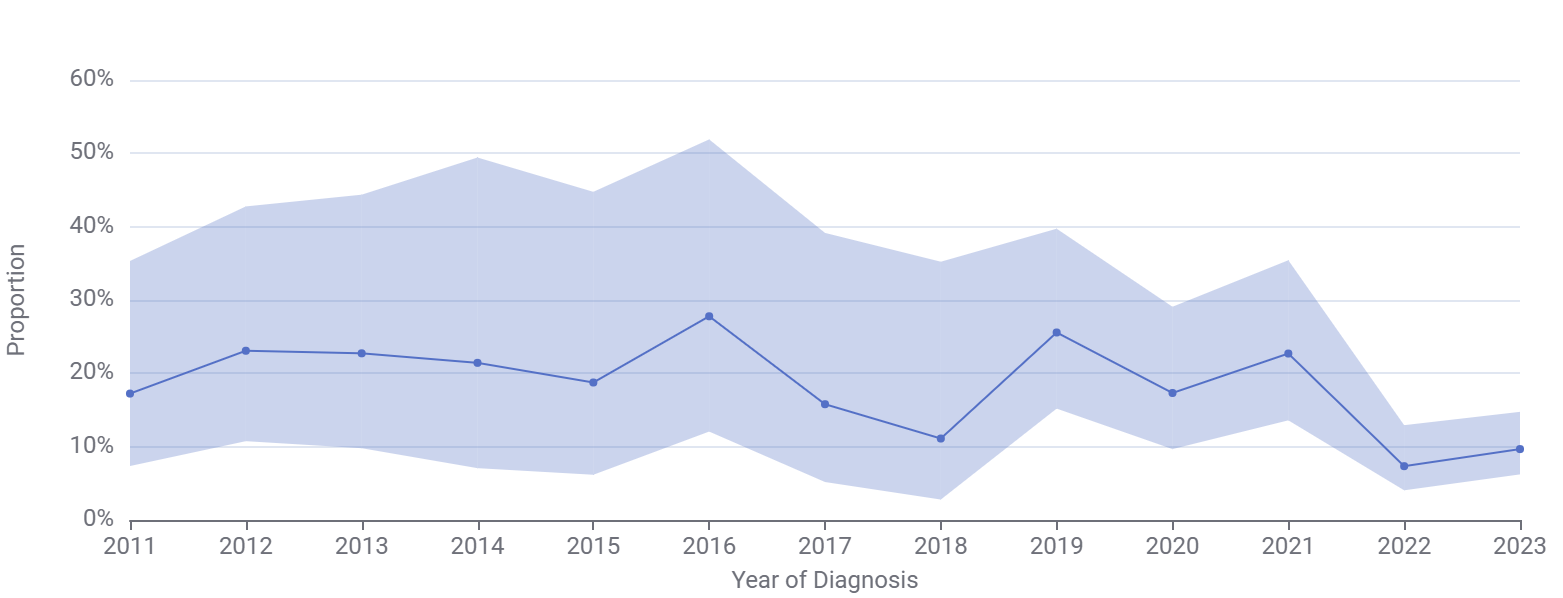
*

*Figure S22. Results of the pre- or post-migration estimation by year of HIV diagnosis.*
